# Expression levels of Fv1: effects on retroviral restriction specificities

**DOI:** 10.1186/s12977-016-0276-7

**Published:** 2016-06-24

**Authors:** Wilson Li, Melvyn W. Yap, Vicky Voss, Jonathan P. Stoye

**Affiliations:** Retrovirus–Host Interactions Laboratory, The Francis Crick Institute, Mill Hill Laboratory, The Ridgeway, Mill Hill, London, NW7 1AA UK; Faculty of Medicine, Imperial College London, London, SW7 2AZ UK

**Keywords:** Fv1, Restriction specificity, Inducible expression, Murine leukaemia virus

## Abstract

**Background:**

The mouse protein Fv1 is a factor that can confer resistance to retroviral infection. The two major Fv1 alleles from laboratory mice, *Fv1*^*n*^ and *Fv1*^*b*^, restrict infection by different murine leukaemia viruses (MLVs). Fv1^n^ restricts B-tropic MLV, but not N-tropic MLV or NB-tropic MLV. In cells expressing Fv1^b^ at natural levels, only N-MLV is restricted, however restriction of NB-MLV and partial restriction of B-MLV were observed when recombinant Fv1^b^ was expressed from an MLV promoter in Fv1 null Mus dunni tail fibroblast cells. To investigate the relationship between expression level and restriction specificity we have developed new retroviral delivery vectors which allow inducible expression of Fv1, and yet allow sufficient production of fluorescent reporter proteins for analysis in our FACS-based restriction assay.

**Results:**

We demonstrated that at concentrations close to the endogenous expression level, Fv1^b^ specifically restricts only N-MLV, but restriction of NB-MLV, and to a lesser extent B-MLV, could be gained by increasing the protein level of Fv1^b^. By contrast, we found that even when Fv1^n^ is expressed at very high levels, no significant inhibition of N-MLV or NB-MLV could be observed. Study of Fv1 mutants using this assay led to the identification of determinants for N/B tropism at an expression level close to that of endogenous Fv1^n^ and Fv1^b^. We also compared the recently described restriction activities of wild mice Fv1 proteins directed against non-MLV retroviruses when expressed at different levels. Fv1 from *M. spretus* restricted N-MLV, B-MLV and equine infectious anaemia virus equally even at low concentrations, while Fv1 from *M. macedonicus* showed even stronger restriction against equine infectious anaemia virus than to N-MLV. Restriction of feline foamy virus by Fv1 of *M. caroli* occurred at levels equivalent to MLV restriction.

**Conclusions:**

Our data indicate that for some but not all Fv1 proteins, gain of restriction activities could be achieved by increasing the expression level of Fv1. However such a concentration dependent effect is not seen with most Fv1s and cannot explain the recently reported activities against non-MLVs. It will be interesting to examine whether overexpression of other capsid binding restriction factors such as TRIM5α or Mx2 result in novel restriction specificities.

**Electronic supplementary material:**

The online version of this article (doi:10.1186/s12977-016-0276-7) contains supplementary material, which is available to authorized users.

## Background

A lengthy period of coevolution between retroviruses and their hosts has resulted in the emergence of numerous host defence mechanisms, as well as viral counter-measures. These include a number of factors such as Fv1, Trim5α, Trim5Cyp and Mx2 that restrict infection through interaction with the retroviral cores [1–4]. Capsid targeting domains of these antiviral proteins define their restriction specificities [5–14]. They have evolved under strong positive selection [10, 15–18], most likely through exposure to endogenous or exogenous retroviruses [8, 15, 19–22]. The study of the mechanisms of restriction is important not only for understanding determinants of viral tropism and species barriers, but also might provide opportunities for the exploitation of these host defence proteins in novel antiretroviral therapies [23, 24].

*Fv1* was first described in the early 1970s as a gene controlling susceptibility of mice to murine leukaemia virus (MLV) [25]. There are two major alleles [26] within inbred laboratory mice, *Fv1*^*n*^ and *Fv1*^*b*^. *Fv1*^*n*^ is present in NIH-Swiss mice, which are permissive to infection by N-tropic MLV (N-MLV), but resistant to infection by B-tropic MLV (B-MLV). In contrast, *Fv1*^*b*^ is expressed in BALB/c mice, which are susceptible to infection by B-MLV but not N-MLV [27, 28]. NB-tropic MLVs (NB-MLVs) such as Moloney MLV can infect cells carrying either *Fv1* allele [29]. The MLV capsid (CA) protein was found to be the target of Fv1 [30, 31], with residue 110 being the major determinant controlling N versus B tropism of MLV [32], while other positions of CA specify NB tropism [7, 30]. Although the precise mechanism of Fv1 restriction is not known, it has been shown that Fv1 blocks at a step after reverse transcription but before integration [33, 34], possibly by preventing the nuclear entry of viral DNA [35]. The Fv1 protein consists of two structural domains: an N-terminal domain (Fv1NTD) and a C-terminal domain (Fv1CTD), joined by a flexible linker [36]. The Fv1NTD, which contains an extended coiled-coil region, forms an antiparallel dimer [36, 37] while the Fv1CTD is believed to be the capsid-targeting domain [5–7, 37]. Fv1^n^ and Fv1^b^ differ only at 3 sites, amino acid (aa) 358, aa 399 and the C-terminus region, all within Fv1CTD [38]. Fv1^n^ encodes K358, V399 and a short 3aa C-terminus, while Fv1^b^ has E358, R399 and a long 22aa C-terminus [38]. Normal levels of Fv1 are low [39] and restriction can be overcome by pre-exposure to MLV [40]. Despite this Fv1 can represent a substantial barrier to MLV-induced leukemogenesis [41].

**Fig. 1:**
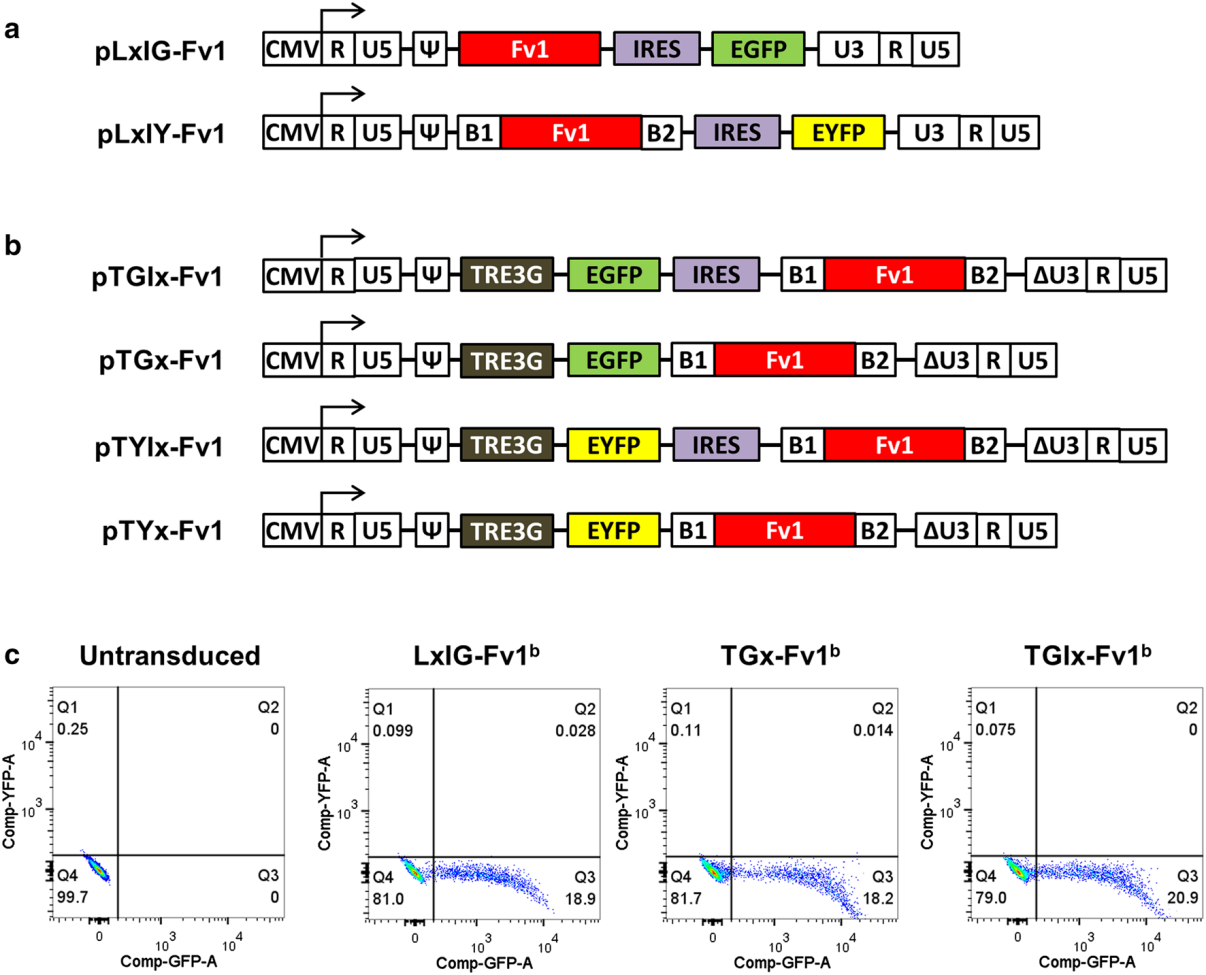
Retroviral vectors used for expression of Fv1. **a** Schematic diagrams showing the plasmids for the previously described non-inducible bicistronic retroviral vectors, LxIG-Fv1 and LxIY-Fv1. After provirus formation, transcription of bicistronic mRNA is driven by the MLV U3 promoter, and translation of Fv1 is initiated at the 5′ CAP while the translation of the fluorescent protein is initiated from an EMCV IRES element. **b** Schematic diagrams showing the plasmids for the novel inducible bicistronic retroviral vectors used in this study. After integration into cells expressing the rtTA3 transactivator, the transcription level of bicistronic RNA driven by the inducible TRE3G promoter is dependent on the concentration of added doxycycline. The translation of the fluorescent protein is initiated at the 5′ CAP. For TGIx-Fv1 and TYIx-Fv1, the translation of Fv1 is initiated from an EMCV IRES. For TGx-Fv1 and TYx-Fv1, the Fv1 ORF is placed downstream to that of the fluorescent protein, and is most likely translated by a ribosome re-initiation mechanism. CMV, Cytomegalovirus immediate early promoter; R, repeated element; U5, unique 5′ element; U3, unique 3′ element; ΔU3, U3 region with deletion in enhancer sequence; Ψ, packaging signal; B1, Gateway cloning attB1 site; B2, Gateway cloning attB2 site; TRE3G, doxycycline-inducible TRE3G promoter. **c** FACS plots showing the separation of GFP+ and GFP− populations by FACS analysis in MDTF-R18 cells transduced with LxIG-Fv1^b^, TGx-Fv1^b^ and TGIx-Fv1^b^ retroviral vectors. Cells transduced with the inducible vectors were treated with 10 μg/mL doxycycline for 24 h prior to analysis

To study the determinants of Fv1 restriction specificity, we developed a two colour FACS assay [5] employing the Mus dunni tail fibroblast (MDTF) cell line, which lacks functional Fv1 due to a premature stop codon in its *Fv1* allele [21, 42]. Transduction of MDTF cells with the retroviral delivery vector LxIG-Fv1 (Fig. 1a) at low multiplicities of infection (MOI) allows the expression of both Fv1 and EGFP in a subpopulation of cells [5]. A bicistronic vector mRNA is constitutively transcribed from the integrated proviral vector genome, allowing the CAP-dependent translation of Fv1 and IRES-dependent translation of EGFP [43]. Mixed populations of cells are then infected with VSV-G packaged MLV tester viruses with Gag and Pol derived from N-MLV, B-MLV or NB-MLV, and a genome that allows the expression of EYFP in infected cells. By comparing the infectivity of EYFP tester virus in the EGFP-positive population with that in the EGFP-negative population, the restriction activity due to Fv1 expression could be measured.

Our initial study showed that the expression of recombinant Fv1^n^ in MDTF cells led to restriction of B-MLV by more than tenfold but no inhibition against N-MLV and NB-MLV [5]. However, although previous studies showed that cells naturally expressing endogenous Fv1^b^ only restrict N-MLV but not NB-MLV or B-MLV [29], the expression of recombinant Fv1^b^ in MDTF cells led to restriction of NB-MLV by fivefold, inhibition of B-MLV by 30 %, in addition to the restriction of N-MLV by more than tenfold [5]. Comparison of Fv1 protein levels in transduced MDTF with cell lines which endogenously express Fv1^n^ (N-3T3) or Fv1^b^ (B-3T3) by semi-quantitative western blot suggested that the Fv1 expression level in transduced MDTF cells far exceeded those in cells naturally expressing Fv1 [39]. The expression of recombinant Fv1^b^ in B-3T3 cells using the LxIG-Fv1 vector also led to stronger restriction of all MLVs [5]. These observations led to the idea that overexpression of Fv1 might reveal additional restriction activities not seen with endogenous levels of Fv1.

Various mutants of Fv1^n^ and Fv1^b^ were studied using the two colour assay, including “mix-and-match” mutants that contain different combinations of sequence from Fv1^n^ or Fv1^b^ at the 3 variable sites [5], mutants with alanine introduced at one or more of the variable sites [6], as well as mutants with the truncation of C-terminus from Fv1^n^ or Fv1^b^ [6]. Among the three sites, position 358 appears to have the strongest effect in determining N/B tropism [5, 6]. All mutants with K358 from Fv1^n^ restrict B-MLV but not N-MLV [5, 6]. In contrast all mutants with E358 from Fv1^b^ or A358 restrict N-MLV, and also restrict B-MLV as long as it does not possess the Fv1^b^ C-terminus [5, 6]. All mutants that restrict NB-MLV contain R399 from Fv1^b^ [5, 6]. While these data suggested that residues at all three variable sites could influence the restriction specificities towards MLVs, it is not known whether all of the restriction activities could still be observed when the Fv1 mutants were expressed at a level closer to the endogenous levels of Fv1^n^ and Fv1^b^, or whether the determinants for MLV tropism at low, endogenous levels are different from those following overexpression.

The presence of a common retroviral motif, the Major Homology Region (MHR) within the *Fv1* gene was in part responsible for the suggestion that Fv1 originated as part of the Gag protein of a murine endogenous retrovirus MERV-L [38, 44], and this MHR motif was found to be essential for MLV restriction and capsid binding activities in Fv1^n^ [1, 6]. Phylogenetic analysis of wild mice *Fv1* sequences suggested that the Fv1 was inserted to ancestor of *Mus* species about 4–7 million years ago [8, 15, 21]. Studies of some of these sequences in MDTF cells transduced with the LxIY-Fv1 vector has led to the identification of novel restriction activities directed against MLV, as well as the lentivirus equine infectious anaemia virus (EIAV) and the spumavirus feline foamy virus (FFV) [8]. Many of the Fv1 specificity determinants identified were found in residues which showed evidence for positive selection [15], suggesting that some of these restriction activities of Fv1 was selected for, possibly from pressure during prior exposure to retroviruses [8]. It is therefore of considerable interest to know whether overexpression might contribute to these novel activities.

To explore the relationship between expression levels and restriction specificity we set out to develop new retroviral delivery vectors which allow inducible expression of Fv1 compatible with our two-colour FACS assay for measurement of retroviral infectivity. This would allow us to ask (1) whether restriction factor overexpression inevitably leads to novel patterns of restriction and (2) whether overexpression was required for the new specificities we had observed in different species of mice [8]. This paper reports the development and use of such a system, in combination with the quantitation of Fv1 protein levels, to examine the restriction activities shown by different *Fv1* alleles at different concentrations of restriction factor.

## Results

### Novel vectors to study restriction activities of Fv1 at different expression levels

To examine the relationship between Fv1 expression levels and restriction specificity, we wanted to study the restriction of different MLVs at different concentrations of Fv1^n^ and Fv1^b^, including both endogenous and overexpression levels, using our two-colour FACS-based restriction assay [5]. We initially attempted to develop vectors allowing the expression of *Fv1* under its natural promoter in MDTF cells, so that the restriction activity of Fv1 at endogenous levels could be studied. We constructed a self-inactivating (SIN) retroviral vector in which the natural promoter of *Fv1* drives the transcription of a bicistronic mRNA with the Fv1 ORF, followed by the EMCV IRES and finally the EYFP ORF. However, the low level of transcription driven by the natural Fv1 promoter does not provide sufficient separation between transduced and untransduced cell populations for a 2-colour FACS assay.

We therefore set out to devise an inducible expression system, using components of the Tet-On 3G system (Clontech) [45, 46] and designed new MLV-based SIN vectors for doxycycline-inducible expression of *Fv1* and either EGFP or EYFP (Fig. 1b). Promoters used in doxycycline-inducible expression system often suffer from “leaky expression” due to constitutive transcription activity from the inducible promoter in the absence of doxycycline. Although the *P*_TRE3G_ promoter from the Tet-On 3G system is less leaky than previous doxycycline-inducible promoters [46, 47], even a low level of transcription may be sufficient to drive Fv1^b^ expression above its endogenous level [39]. We therefore modified both transcriptional and translational signals of our previous retroviral vectors, to minimise uninduced Fv1 expression and to maximise the expression level of the reporter gene allowing sufficient separation for FACS analysis.

Previous studies using reporter genes have found that in a bicistronic mRNA with the arrangement of ORF1-IRES-ORF2, the translation of the second ORF is often less efficient than the first ORF [48, 49]. Moreover, an eightfold lower translation efficiency of the second ORF compared to the first ORF has been reported when an attenuated form of EMCV IRES is used [49]. We therefore constructed the TGIx-Fv1 vector, in which the *P*_TRE3G_ promoter drives the transcription of a bicistronic EGFP-IRES-Fv1 mRNA (Fig. 1b). To achieve optimal translation of EGFP through a CAP-dependent initiation mechanism we placed the EGFP close to the 5′ CAP structure of the mRNA, and included a strong Kozak sequence (GCCGCCATGG) for the EGFP ORF [50]. Translation of Fv1 was attenuated by using an IRES with a suboptimal A7 sequence at the bifurcation loop [49] and by including a 71nt sequence (mostly from the Gateway cloning *attB1* recombination site) between the IRES and the Fv1ORF.

We also explored the possibility of reducing the translation of Fv1 by using an upstream ORF in the absence of a functional IRES. There are many examples in which the translation of a downstream ORF is inhibited by the presence of one or more upstream ORFs [51–53]. Of these, most could be explained by an inefficient ribosome reinitiation mechanism, in which following translational termination of the first ORF, the 40S ribosome remains bound to the mRNA and continues scanning it until the reinitiation of translation at the start codon of the downstream ORF [53]. In a study involving IRES-free bicistronic mRNAs each with two reporter genes, the expression level of the second ORF was reported to be up to 1000-fold lower that of the first ORF [48]. Aiming to provide further translational attenuation of Fv1, we therefore constructed the IRES-free TGx-Fv1 vector, in which the EGFP ORF is placed upstream of the Fv1 ORF (Fig. 1b). Since ribosome re-initiation is much less efficient than IRES-mediated initiation, we expected the TGx-Fv1 vector to promote an even lower Fv1 expression level than the TGIx-Fv1 vector in the absence of doxycycline, while retaining optimal expression of EGFP with both vectors in the presence of doxycycline by a CAP-dependent initiation mechanism. EYFP-expressing versions of the inducible vectors, TYIx-Fv1 and TYx-Fv1, were constructed in a similar fashion (Fig. 1b).

 We tested the new inducible vectors in the MDTF-R18 cell line, which constitutively expresses the rtTA3 transactivator [45]. It was derived from MDTF cells transduced with a lentiviral vector encoding rtTA3 and blastocidin resistance and selected from a large number of antibiotic resistant clones for strong doxycycline induction of the *P*_TRE3G_ promoter and minimal leakiness in the absence of doxycycline (Additional file 1). However, initial titration experiments showed that TGx-Fv1 transduced MDTF-R18 cells required induction for 24 h with at least 100 ng/mL of doxycycline to allow the FACS separation of transduced and untransduced populations (Additional file 2). We therefore modified our standard Fv1 restriction assay [5] accordingly. Fv1 expression in delivery virus (TGx-Fv1 or TGIx-Fv1) transduced cells was induced with varying concentrations of doxycycline (0–1000 ng/mL) for 24 h before infection with EYFP tester virus. 24 h after tester virus infection, during which time Fv1-mediated restriction would be predicted to occur, 10 µg/mL of doxycycline was added to all samples to ensure a sufficient EGFP expression for the two-colour FACS assay. Using this approach, the GFP separation of cells transduced with either of the inducible vectors was comparable to that of the non-inducible vector pLxIG (Fig. 1c). When doxycycline was first added 24 h after the infection of N-MLV tester virus, no restriction by Fv1^b^ could be detected (Additional file 3), supporting the hypothesis that any Fv1 produced after 24hpi does not interact with the tester virus. This assay now allows us to compare the infectivity of EYFP tester virus in the Fv1-expressing transduced population to that in the control untransduced population, permitting the measurement of Fv1-specific inhibition independent of non-specific effects such as the presence of doxycycline.

### MLV restriction activities of Fv1^n^ and Fv1^b^ at different expression levels

To test the utility of these novel inducible vectors in restriction assays, we measured the MLV restriction activities of Fv1^n^ and Fv1^b^ using N-MLV, B-MLV and NB-MLV with TGIx-Fv1, TGx-Fv1 and LxIG-Fv1 vectors (Table 1). As expected, the results from restriction studies using the LxIG-Fv1 vector in MDTF-R18 cells were similar to those reported previously in MDTF cells [5]. At the same time we tested the restriction activities seen when the TGx-Fv1 and TGIx-Fv1 vectors in cells treated with or without 1000 ng/mL doxycycline for 24 h before infection (Table 1). When using the TGx-Fv1 vector in the absence of doxycycline, neither Fv1^n^ nor Fv1^b^ demonstrated any detectable inhibition of MLV. The complete lack of inhibition suggested that with this vector in the absence of doxycycline both Fv1^n^ and Fv1^b^ were expressed at lower levels than seen in NIH-3T3 or BALB-3T3 (endogenous levels). When treated with doxycycline, the TGx vector-expressed Fv1^n^ restricted B-MLV by fourfold and the Fv1^b^ restricted N-MLV by fivefold. Weak inhibition of NB-MLV by Fv1^b^ by about 20 % was also observed under these conditions. When using the TGIx-Fv1 vector in the absence of doxycycline, partial restriction could be detected with both Fv1^n^ and Fv1^b^, suggesting that the expression levels of Fv1^n^ and Fv1^b^ under these conditions are below their endogenous levels, but substantially higher that in TGx-Fv1 without doxycycline. When treated with doxycycline, the restriction activities against B-MLV and against N-MLV by TGIx-Fv1 appeared substantially stronger than TGx-Fv1, but still weaker than LxIG-Fv1. At this level, partial restriction of NB-MLV by Fv1^b^ was clearly observed. Therefore, across the five induction levels, the strength of each MLV restriction activity could be arranged, from the weakest to the strongest, in the order of TGx-Fv1 −Dox < TGIx-Fv1 −Dox < TGx-Fv1 +Dox < TGIx-Fv1 +Dox < LxIG-Fv1.

**Table 1:** Restriction activity of Fv1^n^ and Fv1^b^ against MLVs at different expression levels

Vector	TGx-Fv1		TGIx-Fv1		LxIG-Fv1
Dox (ng/mL)	0	1000	0	1000	0
Fv1^n^					
N-MLV	1.15 ± 0.07	1.16 ± 0.04	1.22 ± 0.11	1.13 ± 0.05	1.07 ± 0.03
B-MLV	1.15 ± 0.09	***0.24*** ***±*** ***0.04***	*0.56* ± *0.01*	***0.14*** ***±*** ***0.02***	***0.06*** ***±*** ***0.01***
NB-MLV	1.17 ± 0.06	1.18 ± 0.04	1.21 ± 0.04	1.09 ± 0.06	1.08 ± 0.01
Fv1^b^					
N-MLV	1.02 ± 0.09	***0.17*** ***±*** ***0.01***	*0.45* ± *0.09*	***0.13*** ***±*** ***0.01***	***0.07*** ***±*** ***0.02***
B-MLV	1.13 ± 0.07	1.10 ± 0.06	1.12 ± 0.02	0.87 ± 0.01	*0.63* ± *0.02*
NB-MLV	1.21 ± 0.04	0.79 ± 0.03	1.01 ± 0.07	*0.35* ± *0.02*	***0.21*** **±** **0.03**

Table shows mean and standard deviation values calculated from three independent restriction experiments carried out in transduced MDTF-R18 cells. A lower restriction ratio indicates stronger inhibition by Fv1. We arbitrarily divide restriction activity into three levels; Full, shown in bolditalic (restriction ratio ≤0.3), Partial, indicated in italics (0.3 < ratio ≤ 0.7) and None (ratio > 0.7)

**Fig. 2:**
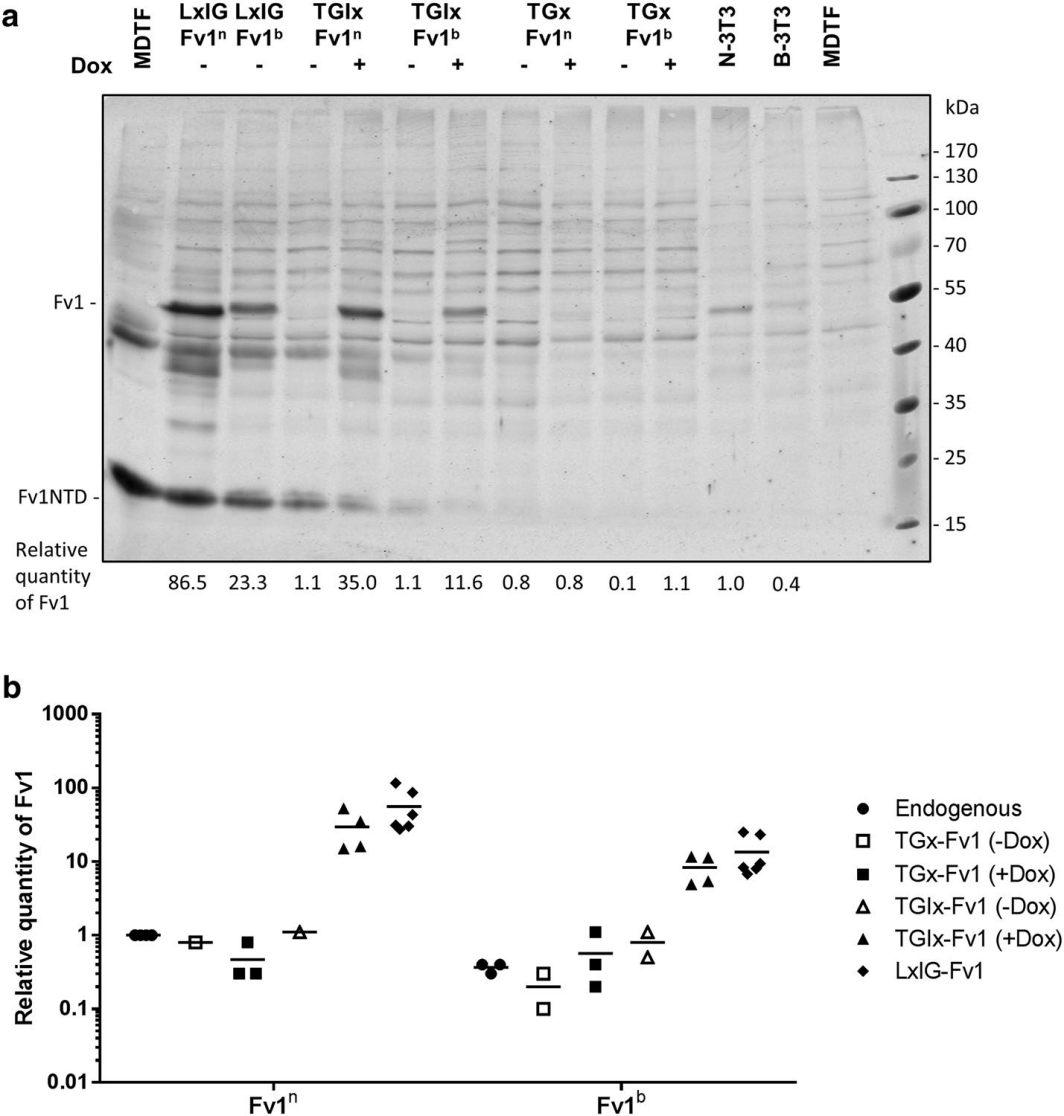
Expression levels of Fv1^n^ and Fv1^b^ from inducible and non-inducible retroviral vectors. **a** Quantitative western blot analysis of Fv1 in N-3T3 cells, B-3T3 cells, and in MDTF-R18 cells transduced with either non-inducible or inducible vectors expressing Fv1 (MOI 0.7). Fv1 was detected using an anti-Fv1 antibody and the LI-COR Odyssey infrared fluorescent western blot system. 1000 ng/mL doxycycline was added to culture media for induced samples. Quantities of Fv1 in lysate samples were interpolated from a standard curve generated using fluorescent signals from known quantities of purified Fv1NTD protein loaded on the same gel. Numbers under the blot indicate the quantities of Fv1 in each sample relative to that in the N-3T3 sample. **b** Quantities of Fv1 in transduced MDTF-R18 and B-3T3, relative to that of N-3T3. Data from Additional file 1 are shown as a grouped scatter plot. Mean values are represented by *horizontal lines*, while individual results are plotted as symbols: *filled circle*, endogenous Fv1 from N-3T3 or B-3T3 cells; *open square*, TGx-Fv1 without dox; *filled square*, TGx-Fv1 with 1000 ng/mL dox; *open triangle*, TGIx-Fv1 without dox; *filled triangle*, TGIx-Fv1 with 1000 ng/mL dox; *filled diamond*, LxIG-Fv1 without dox

Since the difference in degree of restriction is most likely due to differences in Fv1 expression levels, we next attempted to compare the Fv1 protein levels under these five conditions with the natural Fv1 levels in N-3T3 and B-3T3 cells in quantitative western blots (Fig. 2; Additional file 4). The expression level of Fv1^n^ in N-3T3 cells was threefold higher than that of Fv1^b^ in B-3T3 cells. The greater mobility of Fv1^n^ compared to Fv1^b^ can be explained by a 19 amino acid difference in length between the two proteins [38]. The LxIG-Fv1 vector gave around 55-fold overexpression of Fv1^n^ and 30-fold overexpression of Fv1^b^. Overexpression of Fv1^n^ and Fv1^b^ were also observed with doxycycline treated TGIx-Fv1 cells, although the expression levels were only about half of that seen with LxIG-Fv1. In both of these overexpression systems, the expression of Fv1^n^ is 3–4 fold higher than that of Fv1^b^ when the same vector and doxycycline concentration were used, presumably as a result of differing protein stability since the non-coding sequences in pairs of vectors are identical. The Fv1 protein levels with TGx-Fv1 −Dox, TGIx-Fv1 −Dox and TGx-Fv1 +Dox were much lower. In some cases, faint bands corresponding to the molecular weight of Fv1 could be observed by fluorescent western blot. However, the weak fluorescent signals from these bands were almost indistinguishable from background noise, and cannot be quantified reliably. Analysis is further complicated by the copy number of the transduced Fv1—haploid when transduced at an MOI of 1 and diploid in N-3T3 and B-3T3 cells. Nevertheless, the western blot results confirmed that at a given doxycycline concentration, a lower Fv1 expression level can be seen using pTGx-Fv1 than TGIx-Fv1. Comparison of these data (Fig. 2; Table 1) with our previous analysis of restriction in N-3T3 and B-3T3 cells [5] suggests that natural Fv1 expression levels lie intermediate between those found with TGx-Fv1 +Dox and TGIx-Fv1 +Dox. Under these conditions Fv1^b^ has little or no effect on B-MLV but significant restriction of NB-MLV.

### MLV restriction activities at “superexpression” levels of Fv1^n^ and Fv1^b^

**Fig. 3:**
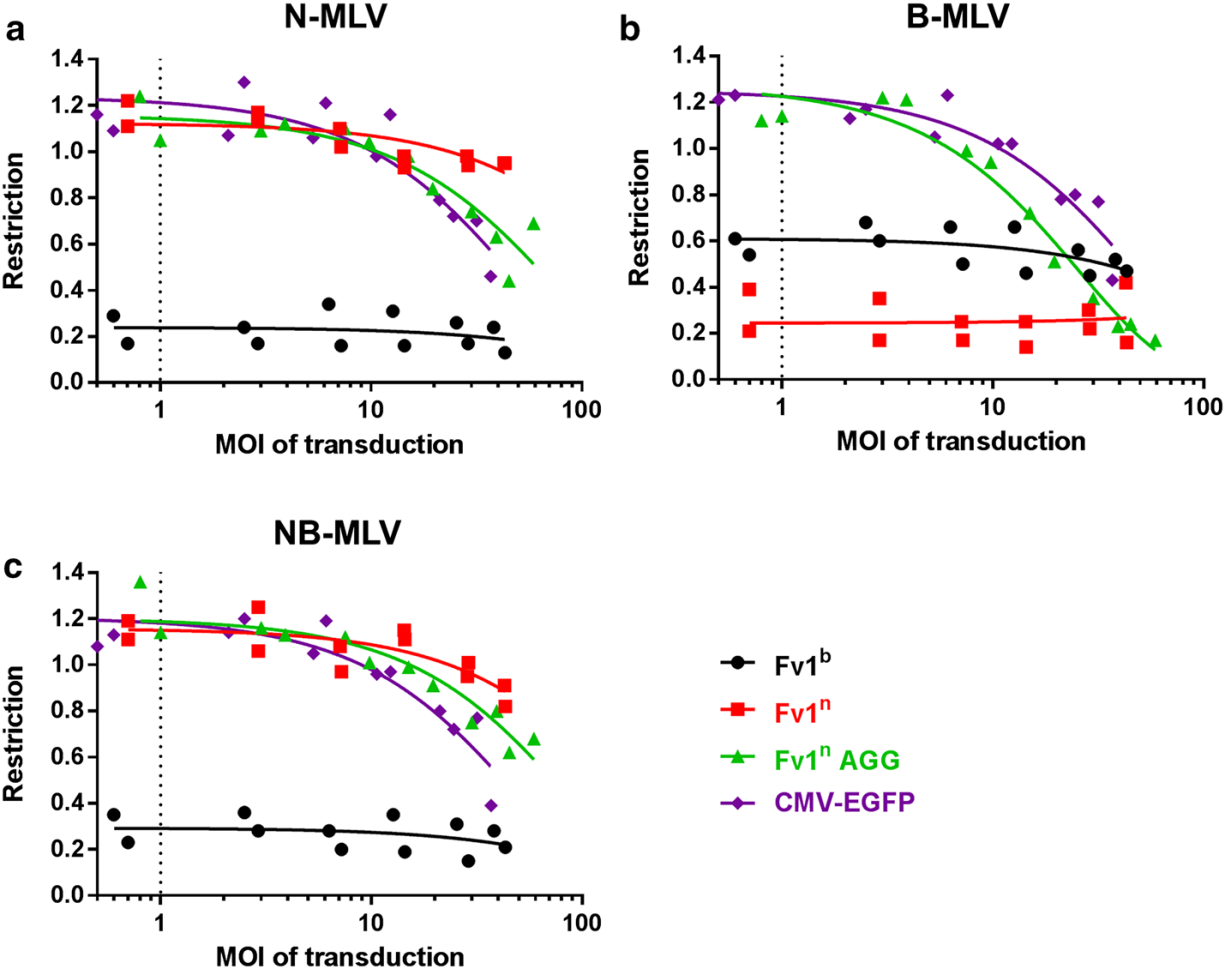
Restriction activity of various Fv1 at “superexpression” levels against different MLVs. Restriction of **a** N-MLV, **b** B-MLV and **c** NB-MLV in cells transduced at various MOIs with the LxIG-Fv1^n^ vector (*red squares*), the LxIG-Fv1^b^ vector (*black circles*), the LxIG-Fv1^n^-AGG mutant vector (*green triangles*) or the EGFP tester virus (*purple diamonds*). *Dotted vertical lines* indicate an MOI of 1

While the overexpression of Fv1^b^ using LxIG-Fv1 enables the restriction of NB-MLV and even partial restriction of B-MLV, the overexpression of Fv1^n^ using LxIG-Fv1 did not lead to even the slightest inhibition of N-MLV or NB-MLV. However it seemed possible that inhibition of N-MLV and NB-MLV by Fv1^n^ might be observed at an expression level even higher than that by the LxIG-Fv1 vector. We therefore set out to express Fv1 at “superexpression” levels by transducing MDTF-R18 cells with the LxIG-Fv1 vector at high MOI’s. Such cells were then mixed with untransduced cells as internal controls, and infected with EYFP tester viruses for two colour restriction assays. As control for non-specific effects of high provirus copy number unrelated to Fv1 expression e.g. EYFP overexpression, two control vectors were employed. The first contains the genome for the generation of EGFP tester virus, in which a CMV promoter drives the expression of EGFP only. The second, LxIG-Fv1^n^-AGG, is a mutated form of the LxIG-Fv1^n^ vector in which the start codon of Fv1 ORF is changed from ATG to AGG to abolish Fv1 expression, but still allow the expression of EGFP using the IRES. Although non-specific restriction was observed at MOI’s greater than ten (Fig. 3), in none of these experiments did LxIG-Fv1^n^ exhibit stronger inhibition of N-MLV or NB-MLV than any of the control vectors, suggesting that further increasing the concentration of Fv1^n^ in cells does not enable the inhibition of N-MLV and NB-MLV by Fv1^n^. In addition no additional restriction of B-MLV by Fv1^b^ was seen at higher MOI’s.

### Restriction studies of Fv1^n^ and Fv1^b^ mutants at different expression levels

Previous restriction studies using the LxIG-Fv1 vector on 6 “mix-and-match” mutants which harbour either Fv1^n^ or Fv1^b^ sequence at positions 358, 399 and at the C-terminus [5], and on Fv1^n^ and Fv1^b^ with truncation at the C-terminus [6], have showed that restriction specificity against different MLVs appeared to be a combinatorial property of C-terminal Fv1 sequences, at least when present at overexpressed levels [5, 6]. We extended the studies of these mutants using the new inducible vectors, to ask whether the novel specificities revealed in those previous studies were dependent upon over expression, focussing our analysis on comparing the MLV restriction activities of endogenous-like level of Fv1 (TGx-Fv1 +Dox and TGIx-Fv1 +Dox) to that of overexpression levels (LxIG-Fv1). In all we tested three different viruses for restriction with eight different Fv1 constructs in addition to the parental Fv1^n^ and Fv1^b^. Examining these 24 combinations (Table 2) revealed that the majority resembled the patterns seen with Fv1^n^; nine showed restriction profiles (profile A) closely resembling those seen for Fv1^n^ against B-MLV while a further nine showed no effect at any Fv1 concentration like Fv1^n^ and N-MLV (profile D). In four cases full restriction was observed but only at high Fv1 concentrations, resembling the effect of Fv1^b^ on NB-MLV (profile B). Two hybrid Fv1s resembled Fv1^b^ by giving partial restriction (by nnb and bb_ on N-MLV and B-MLV, respectively) at high concentrations (profile C).

**Table 2:** Restriction activity of various Fv1 mutants against MLVs at different Fv1 expression levels

Vector	TGx-Fv1	TGIx-Fv1	TGx-Fv1	TGIx-Fv1	LxIG-Fv1	Activity profile^a^
Dox (ng/mL)	0	0	1000	1000	0	
Fv1^n^						
N-MLV	1.15 ± 0.07	1.22 ± 0.11	1.16 ± 0.04	1.13 ± 0.05	1.07 ± 0.03	D
B-MLV	1.15 ± 0.09	*0.56* ± *0.01*	***0.24*** ***±*** ***0.04***	***0.14*** ***±*** ***0.02***	***0.06*** ***±*** ***0.01***	A
NB-MLV	1.17 ± 0.06	1.21 ± 0.04	1.18 ± 0.04	1.09 ± 0.06	1.08 ± 0.01	D
Fv1^b^						
N-MLV	1.02 ± 0.09	*0.45* ± *0.09*	***0.17*** ***±*** ***0.01***	***0.13*** ***±*** ***0.01***	***0.07*** ***±*** ***0.02***	A
B-MLV	1.13 ± 0.07	1.12 ± 0.02	1.10 ± 0.06	0.87 ± 0.01	*0.63* ± *0.02*	C
NB-MLV	1.21 ± 0.04	1.01 ± 0.07	0.79 ± 0.03	*0.35* ± *0.02*	***0.21*** ***±*** ***0.03***	B
Fv1bbn						
N-MLV	0.97 ± 0.02	*0.38* ± *0.04*	***0.17*** ***±*** ***0.06***	***0.11*** ***±*** ***0.00***	***0.06*** ***±*** ***0.01***	A
B-MLV	1.10 ± 0.02	*0.58* ± *0.10*	***0.29*** ***±*** ***0.06***	***0.11*** ***±*** ***0.02***	***0.07*** ***±*** ***0.03***	A
NB-MLV	0.96 ± 0.08	*0.33* ± *0.07*	***0.19*** ***±*** ***0.04***	***0.08*** ***±*** ***0.02***	***0.08*** ***±*** ***0.02***	A
Fv1nnb						
N-MLV	1.18 ± 0.06	1.09 ± 0.07	1.07 ± 0.07	0.72 ± 0.10	*0.58* ± *0.04*	C
B-MLV	1.13 ± 0.09	0.76 ± 0.03	*0.52* ± *0.03*	***0.14*** ***±*** ***0.03***	***0.09*** ***±*** ***0.02***	A
NB-MLV	1.15 ± 0.10	1.16 ± 0.06	1.07 ± 0.10	1.07 ± 0.13	1.07 ± 0.05	D
Fv1bnn						
N-MLV	1.14 ± 0.07	*0.46* ± *0.01*	***0.18*** ***±*** ***0.07***	***0.12*** ***±*** ***0.02***	***0.09*** ***±*** ***0.06***	A
B-MLV	1.20 ± 0.01	1.04 ± 0.04	0.93 ± 0.05	*0.33* ± *0.03*	***0.12*** ***±*** ***0.03***	B
NB-MLV	1.21 ± 0.12	1.09 ± 0.10	1.12 ± 0.06	1.02 ± 0.03	0.98 ± 0.05	D
Fv1nbb						
N-MLV	1.17 ± 0.04	1.17 ± 0.02	1.05 ± 0.07	1.01 ± 0.03	0.93 ± 0.07	D
B-MLV	1.18 ± 0.04	0.81 ± 0.08	*0.66* ± *0.03*	***0.12*** ***±*** ***0.05***	***0.07*** ***±*** ***0.02***	A
NB-MLV	1.15 ± 0.06	1.09 ± 0.05	1.12 ± 0.11	1.17 ± 0.07	1.10 ± 0.07	D
Fv1nbn						
N-MLV	1.20 ± 0.05	1.10 ± 0.03	1.12 ± 0.09	1.03 ± 0.07	1.02 ± 0.11	D
B-MLV	1.12 ± 0.07	*0.50* ± *0.04*	***0.23*** ***±*** ***0.01***	***0.09*** ***±*** ***0.02***	***0.08*** ***±*** ***0.03***	A
NB-MLV	1.11 ± 0.02	*0.62* ± *0.11*	*0.33* ± *0.05*	***0.12*** ***±*** ***0.03***	***0.09*** ***±*** ***0.02***	A
Fv1bnb						
N-MLV	1.05 ± 0.08	*0.56* ± *0.06*	***0.22*** ***±*** ***0.03***	***0.10*** ***±*** ***0.02***	***0.06*** ***±*** ***0.01***	A
B-MLV	1.09 ± 0.05	1.13 ± 0.02	1.08 ± 0.08	1.12 ± 0.10	1.11 ± 0.04	D
NB-MLV	1.13 ± 0.07	1.09 ± 0.09	1.08 ± 0.11	1.09 ± 0.05	1.12 ± 0.08	D
Fv1nn_						
N-MLV	1.17 ± 0.04	1.15 ± 0.04	1.09 ± 0.07	1.18 ± 0.05	1.10 ± 0.06	D
B-MLV	1.12 ± 0.11	1.11 ± 0.04	1.07 ± 0.02	*0.37* ± *0.05*	***0.09*** ***±*** ***0.02***	B
NB-MLV	1.12 ± 0.10	1.11 ± 0.05	1.12 ± 0.13	1.05 ± 0.06	1.11 ± 0.05	D
Fv1bb_						
N-MLV	1.07 ± 0.17	1.04 ± 0.05	1.07 ± 0.04	***0.15*** ***±*** ***0.04***	***0.06*** ***±*** ***0.02***	B
B-MLV	1.14 ± 0.04	1.15 ± 0.05	1.09 ± 0.11	0.77 ± 0.04	***0.22*** ***±*** ***0.01***	C
NB-MLV	1.13 ± 0.03	1.04 ± 0.06	1.07 ± 0.10	***0.20*** ***±*** ***0.01***	***0.05*** ***±*** ***0.02***	B

We arbitrarily divide restriction activity into three levels; Full, shown in bolditalic (restriction ratio ≤0.3), Partial, indicated in italics (0.3 < ratio ≤ 0.7) and None (ratio > 0.7)

Table shows mean and standard deviation values calculated from three independent restriction experiments carried out in transduced MDTF-R18 cells. Data for Fv1^n^ and Fv1^b^ from Table 1

^a^Classification into groups of constructs with similar activity profiles. A. Full restriction; B. Full restriction only at elevated concentration; C. Partial restriction at high concentrations; D. No restriction. Full and partial restriction defined as in Table 1

**Fig. 4:**
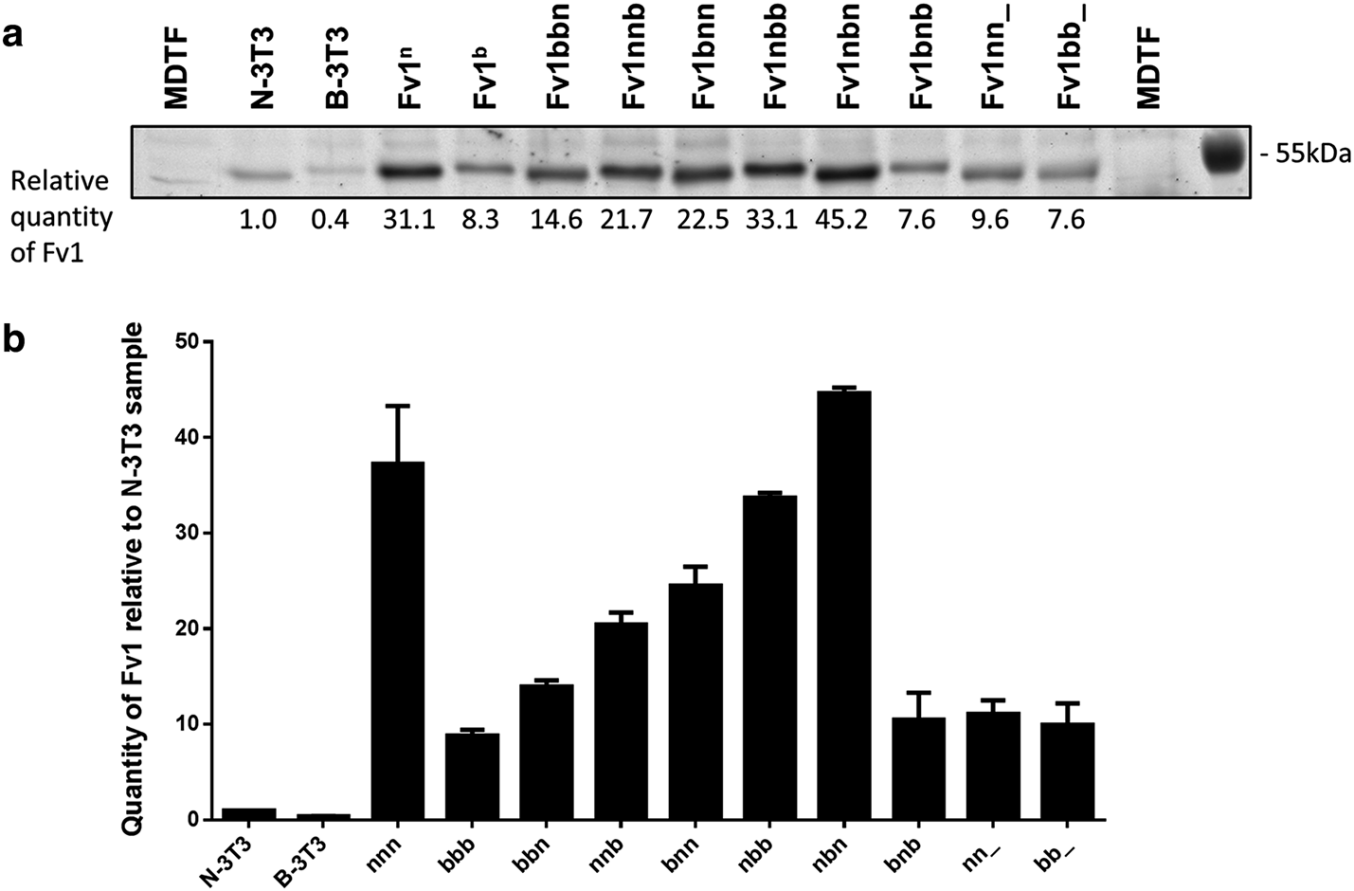
Comparison of endogenous and overexpression levels of Fv1^n^ and Fv1^b^. **a** Quantitative western blot analysis of Fv1 in N-3T3, B-3T3 and MDTF transduced with LxIG-Fv1 deliver vectors containing wild type or mutant Fv1 ORF. Fv1 was detected using an anti-Fv1 antibody and the LI-COR Odyssey infrared fluorescent western blot system. Quantities of Fv1 in lysate samples were interpolated from a standard curve generated using fluorescent signals from known quantities of purified Fv1NTD protein loaded on the same gel. *Numbers* under the blot indicate the quantities of Fv1 in each sample relative to that in the N-3T3 sample. **b** A *bar chart* showing the mean and mean deviation values from two independent quantitation experiments

To test for possible effects on protein stability of permuting the different Fv1 alleles we compared the protein levels of the different Fv1 chimeras (Fig. 4). No single change explained the difference in expression levels of the different alleles of Fv1. All chimeras have a protein level between that of Fv1^n^ and Fv1^b^ with the sole exception of Fv1nbn that has a higher protein level than Fv1^n^. Only in one case out of five in which “high induction” conditions are required for full restriction protein concentration (nn_ compared to Fv1^n^) can a reduction in protein concentration explain the need for higher expression. In the remaining cases, e.g. bb_ compared to Fv1^b^, protein expression is at least as high in the new construct as in the parental allele (Fig. 4) while more Fv1 as judged by induction conditions is required for restriction (Table 2).

### Retroviral restriction activities of wild mice Fv1 at different expression levels

We have previously reported restriction activities against EIAV and FFV of Fv1 alleles from wild mice, identified using the non-inducible vector LxIY-Fv1 (EYFP version of LxIG-Fv1, Fig. 1a) in our two colour restriction assay [8]. Although these novel restriction activities appeared to be equally strong as those against MLV under conditions of overexpression, it is possible that they represent consequences of overexpression. For Fv1 from wild mice, the natural expression levels are not known due to the lack of cell lines derived from these mice. We therefore compared the retroviral restriction activities and expression levels of selected Fv1 alleles (Fv1^b^, Fv1MIN2, Fv1SPR1, Fv1MAC and Fv1CAR1) at different induction levels using the pTYIx-Fv1 vector, to gauge the strength of these restriction activities. Fv1MIN2 from *M. minutoides* was selected because while some studies suggested that this Fv1 allele restricts N-MLV and B-MLV but not NB-MLV [15, 54], our previous restriction analysis suggested that it also restricts NB-MLV [8]. Fv1SPR1 (from *M. spretus*), Fv1MAC (from *M. macedonicus*) and Fv1CAR1 (from *M. caroli*) were chosen on the basis of restriction activities against non-gammaretroviruses [8].

We used the TYIx-Fv1 vector together with the non-inducible LxIY-Fv1 vector to allow the expression of Fv1 at a wide range of concentrations ranging from sub-endogenous to overexpression, for analysis of restriction activities against MLV, EIAV and FFV after treating cells with different amount of doxycycline (Table 3). At 10 ng/mL doxycycline, the restriction phenotype of Fv1^b^ appears to be closest to that of endogenous Fv1^b^, with full restriction of N-MLV and minimal inhibition of NB-MLV. At this induction level, some restriction activities that appeared strong when Fv1 was overexpressed become significantly weaker (e.g. NB-MLV inhibition by Fv1^b^ and Fv1MIN2), while other activities remain strong at this induction level (e.g. N-MLV restriction by Fv1^b^ and Fv1MIN2). These restriction data from multiple expression levels allowed us to compare the strength of restriction activities of the same Fv1 against different retroviruses. Consistent with earlier data (Table 1), the restriction activity of Fv1^b^ appeared to be strongest against N-MLV, moderate against NB-MLV, and weakest against B-MLV. In contrast, Fv1MIN2 appears to restrict both N-MLV and B-MLV strongly, and show weaker restriction against NB-MLV. Fv1SPR1 had similar restriction activities against N-MLV, B-MLV and EIAV even at low expression levels. Fv1MAC showed stronger restriction activity against EIAV than against N-MLV.

**Table 3:** Restriction activity of selected Fv1 alleles against different retroviruses at different Fv1 expression levels

Vector	TYIx-Fv1				LxIY-Fv1
Dox (ng/mL)	0	10	100	1000	0
Fv1^b^					
N-MLV	*0.46* ± *0.02*	**0** ***.21*** ***±*** ***0.01***	***0.14*** ***±*** ***0.02***	***0.13*** ***±*** ***0.02***	***0.09*** ***±*** ***0.01***
B-MLV	1.09 ± 0.05	0.96 ± 0.05	0.83 ± 0.05	0.77 ± 0.03	*0.48* ± *0.02*
NB-MLV	0.93 ± 0.06	*0.51* ± *0.03*	*0.35* ± *0.01*	*0.33* ± *0.04*	***0.16*** ***±*** ***0.01***
EIAV	n.d.	n.d.	n.d.	n.d.	*0.52* ± *0.01*
FFV	n.d.	n.d.	n.d.	n.d.	1.00 ± 0.02
Fv1MIN2					
N-MLV	*0.43* ± *0.02*	***0.18*** ***±*** ***0.02***	***0.14*** ***±*** ***0.02***	***0.11*** ***±*** ***0.02***	***0.04*** ***±*** ***0.01***
B-MLV	*0.33* ± *0.01*	***0.16*** ***±*** ***0.03***	***0.12*** ***±*** ***0.01***	***0.11*** ***±*** ***0.02***	***0.04*** ***±*** ***0.01***
NB-MLV	1.02 ± 0.04	*0.52* ± *0.02*	***0.27*** ***±*** ***0.03***	***0.23*** ***±*** ***0.05***	***0.10*** ***±*** ***0.01***
EIAV	n.d.	n.d.	n.d.	n.d.	1.08 ± 0.04
FFV	n.d.	n.d.	n.d.	n.d.	1.00 ± 0.02
Fv1SPR1					
N-MLV	*0.70* ± *0.04*	***0.28*** ***±*** ***0.01***	***0.17*** ***±*** ***0.02***	***0.16*** ***±*** ***0.02***	***0.12*** ***±*** ***0.01***
B-MLV	0.75 ± 0.04	*0.31* ± *0.05*	***0.19*** ***±*** ***0.01***	***0.19*** ***±*** ***0.02***	***0.13*** ***±*** ***0.01***
NB-MLV	n.d.	n.d.	n.d.	n.d.	1.17 ± 0.01
EIAV	*0.65* ± *0.06*	*0.32* ± *0.03*	***0.24*** ***±*** ***0.02***	***0.24*** ***±*** ***0.02***	***0.20*** ***±*** ***0.01***
FFV	n.d.	n.d.	n.d.	n.d.	1.02 ± 0.01
Fv1MAC					
N-MLV	1.10 ± 0.04	*0.70* ± *0.02*	*0.35* ± *0.02*	***0.30*** ***±*** ***0.02***	***0.18*** ***±*** ***0.01***
B-MLV	1.08 ± 0.09	1.08 ± 0.02	1.10 ± 0.06	1.02 ± 0.02	1.24 ± 0.02
NB-MLV	n.d.	n.d.	n.d.	n.d.	1.24 ± 0.01
EIAV	0.79 ± 0.02	*0.44* ± *0.02*	***0.27*** ***±*** ***0.02***	***0.26*** ***±*** ***0.03***	***0.21*** ***±*** ***0.01***
FFV	n.d.	n.d.	n.d.	n.d.	1.01 ± 0.03
Fv1CAR1					
N-MLV	n.d.	n.d.	n.d.	n.d.	1.21 ± 0.07
B-MLV	n.d.	n.d.	n.d.	n.d.	1.15 ± 0.03
NB-MLV	n.d.	n.d.	n.d.	n.d.	1.17 ± 0.01
EIAV	n.d.	n.d.	n.d.	n.d.	1.17 ± 0.09
FFV	0.95 ± 0.01	*0.40* ± *0.04*	***0.18*** ***±*** ***0.02***	***0.18*** ***±*** ***0.03***	***0.13*** ***±*** ***0.01***

We arbitrarily divide restriction activity into three levels; Full, shown in bolditalic (restriction ratio ≤0.3), Partial, indicated in italics (0.3 < ratio ≤ 0.7) and None (ratio > 0.7)

Table shows the results from this study using the TYIx-Fv1 inducible vector, with mean and standard deviation values calculated from two independent duplicate experiments. The last column shows results from our previous study using the non-inducible LxIY-Fv1 vector in MDTF cells [8]. n.d., not determined

**Fig. 5:**
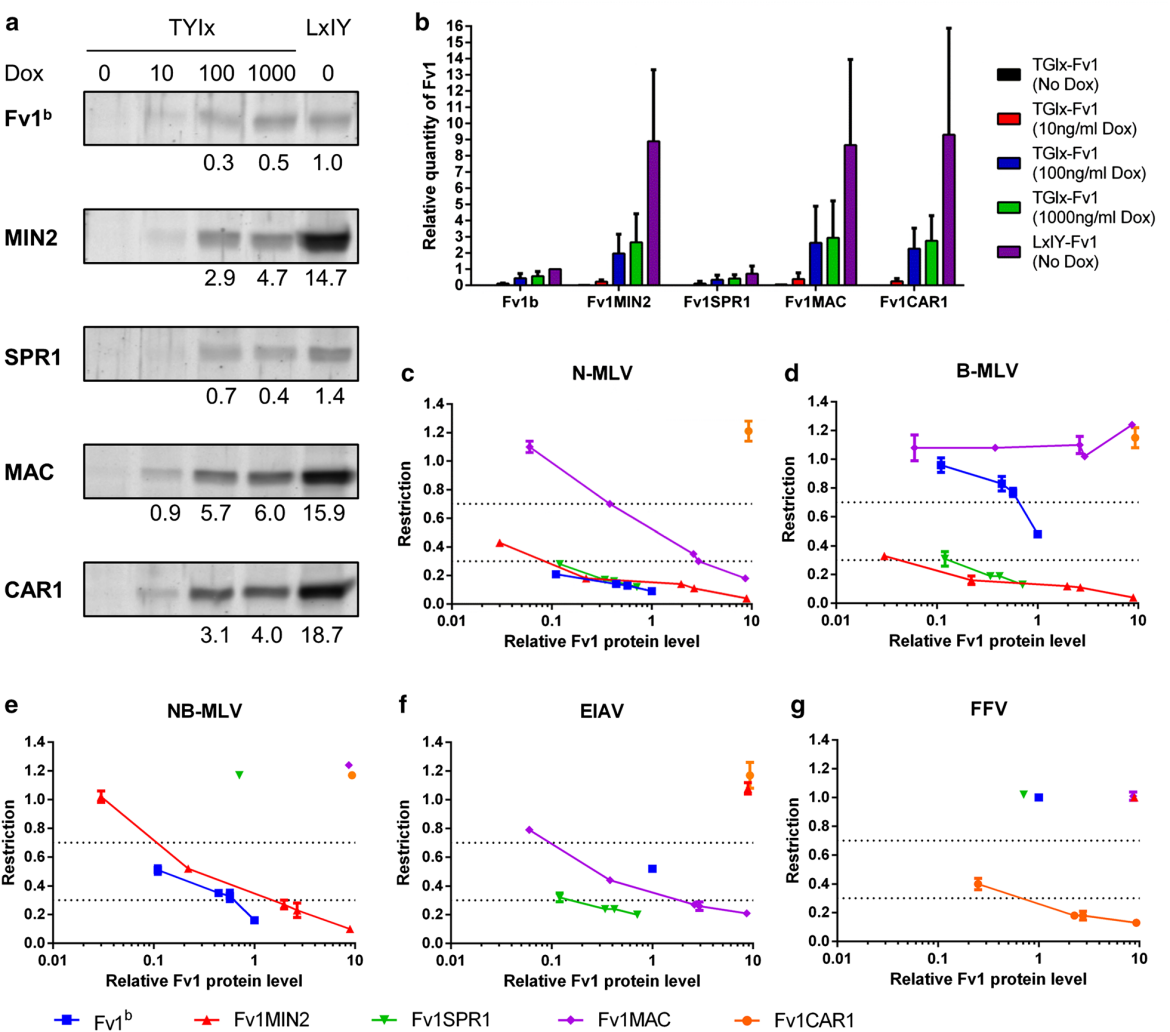
Restriction activity and expression levels of selected Fv1 alleles. **a** Quantitative western blot analysis of various Fv1 alleles in MDTF-R18 cells transduced with either TYIx-Fv1 or LxIY-Fv1 vectors. Fv1 was detected using an anti-Fv1 antibody and the LI-COR Odyssey infrared fluorescent western blot system. Transduced cells were treated with 0, 10, 100, or 1000 ng/mL doxycycline for 24 h before challenging with EGFP-expressing tester viruses. Quantities of Fv1 in lysate samples were interpolated from a standard curve generated using fluorescent signals from known quantities of purified Fv1NTD protein loaded on the same gel. Numbers under the blot indicate the quantities of Fv1 in each sample relative to that in the LxIY-Fv1^b^ sample. **b** Graph showing the mean and standard deviation values of relative quantities of selected Fv1 alleles in MDTF-R18 cells transduced with TYIx-Fv1 or LxIY-Fv1 vector relative to that in the LxIY-Fv1^b^ sample. Two repeated quantitative western blots were carried for each of two samples of transduced cells, and the results are also listed in Table S2. Cells transduced with TYIx-Fv1 vectors were treated with 0 (*black*), 10 (*red*), 100 (*blue*) or 1000 (*green*) ng/mL dox for 24 h. Cells transduced with LxIY-Fv1 vectors (*purple*) were not treated with dox. **c**–**g** Scatter plots showing mean values and standard deviation of **c** N-MLV, **d** B-MLV, **e** NB-MLV, **f** EIAV and **g** FFV restriction activities from Table 2 against mean relative Fv1 protein levels from Fig. 3b. *Blue squares*, Fv1^b^; *red upright triangles*, Fv1MIN2; *green inverted triangles*, Fv1SPR1; *purple diamonds*, Fv1MAC; *orange circles*, Fv1CAR1. The *two dotted horizontal lines* indicate the boundaries between full restriction and partial restriction (0.7), and between partial restriction and no restriction (0.3)

Since the expression level of different Fv1s can be rather different at the same induction level (Fig. 2), we analysed the Fv1 protein levels in all five sets of conditions (Fig. 5a, b; Additional file 5). Indeed, there are large differences in the protein levels of different Fv1s at the same concentration of added doxycycline. The five Fv1s could be divided into two groups based on their expression levels. When expressed from the pLxIY vector, average protein levels of Fv1MIN2, Fv1MAC and Fv1CAR1 were ninefold higher than that of Fv1^b^ and Fv1SPR1 (Fig. 5b). This was even higher than seen with Fv1^n^, which was fourfold higher than Fv1^b^ (Fig. 2). To compare the restriction activities of different Fv1s against the same virus, we plotted the Fv1 restriction activities against the Fv1 protein levels (Fig. 5c–g). At all Fv1 protein levels, the restriction of N-MLV by Fv1^b^, Fv1MIN2 and Fv1SPR1 was similar but much stronger than that of Fv1MAC. The quantity of Fv1 required to fully restrict N-MLV (restriction ratio of ≤0.3) was approximately 30-fold higher for Fv1MAC than that required for Fv1^b^, Fv1MIN2 and Fv1SPR1 (Fig. 5c). Among the non-MLV restriction activities the restriction of EIAV by Fv1SPR1 was also stronger than that of Fv1MAC, with about 20-fold more Fv1MAC required for full restriction of EIAV compared to Fv1SPR1 (Fig. 5f). Additionally, the restriction of EIAV by Fv1MAC and the restriction of FFV by Fv1CAR1 appeared to be weaker and similar to the NB-MLV restriction activity by Fv1^b^ (Fig. 5e–g). In contrast, Fv1SPR1 exhibited strong EIAV restriction activity when expressed at levels comparable to those required for full restriction of N-MLV restriction by Fv1^b^, Fv1MIN2 and Fv1SPR1. In this case at least the novel restriction activity does not require overexpression.

## Discussion

We have developed new retroviral delivery vectors that allow the inducible expression of restriction factors, while simultaneously allowing use of the two colour FACS assay so that reduction in infectivity of tester virus can be compared to that of an internal control. Using these vectors, we examined the relationship between Fv1 overexpression and the gain of additional restriction activities. Our studies provided definitive evidence that NB-MLV restriction and partial restriction of B-MLV could be achieved by overexpressing Fv1^b^, whereas overexpression of Fv1^n^ does not lead to inhibition of N-MLV and NB-MLV. We suggest that the difference in the ability to gain additional restriction activities could be explained the differences in relative binding affinities towards different MLVs. In our previous binding study, we showed specific binding of Fv1^n^ to polymerised CA of B-MLV, but not N-MLV or NB-MLV [1]. In contrast, we saw essentially no difference in binding of Fv1^b^ to polymerised CA of N-MLV, B-MLV and NB-MLV [1]. Subsequent binding studies at different concentration of Fv1^b^ all showed similar binding of Fv1^b^ to CA from all MLVs (Wilson Li, PhD thesis University College London). These data would indicate that whereas Fv1^n^ has much stronger affinity for CA of B-MLV compared to CA of N-MLV and NB-MLV, the differences with Fv1^b^ are much smaller. As a result, the slightly weaker affinities of Fv1^b^ for NB-MLV and B-MLV can be overcome by overexpression but similar levels of overexpression would not be sufficient for detectable inhibition of N-MLV and NB-MLV by Fv1^n^. Differential binding of Fv1^b^ to CA of different MLVs may only be observable at very low Fv1 concentrations, where the amount of bound Fv1^b^ would be below the detection limit of the binding assay.

At induction levels of Fv1^b^ where full restriction of N-MLV was observed, NB-MLV was inhibited by at least 20 % (Tables 1, 2). This would suggest that even at endogenous levels, Fv1^b^ would weakly inhibit NB-MLV. Previous data from B-3T3 cells overexpressing Fv1^n^ are consistent with this idea [5]. In that study it was shown that overexpression of one Fv1 allele in cells with endogenous expression of another Fv1 allele resulted in the restriction phenotype of the overexpressed Fv1, and abolition of the endogenous phenotype [5]. In experiments in which B-3T3 cells were transduced with the delivery vector LxIG-Fv1^n^, cells expressing exogenous Fv1^n^ showed a 20 % increase in the number of NB-tropic MLV compared to non-transduced controls [5], suggesting a level of inhibition by exogenously expressed Fv1^b^ similar to that observed at low induction levels using the inducible vectors.

The protein expression levels of Fv1 vary from allele to allele (Figs. 2, 4). Thus, Fv1^n^, Fv1MAC, Fv1CAR1 and Fv1MIN2 are present at much higher levels than Fv1^b^ and Fv1SPR1 (Fig. 5). The difference between Fv1^n^ and Fv1^b^ is independent of transcription level (Fig. 4) suggesting differential protein stability. This cannot be explained in terms of the individual amino acid differences between the two proteins (Fig. 4). Although sequences at positions 358, 399 and the C-terminus could affect the overexpression levels of Fv1^n^ and Fv1^b^ (Fig. 4), individual residues at these positions could not explain the overexpression level of wild mice Fv1s. For example, both Fv1MAC and Fv1SPR1 have the Fv1^n^ residues K358 and V399, and Fv1^b^-like long C-termini [8], but the expression levels of Fv1MAC were much higher than those of Fv1SPR1 (Fig. 5). It is conceivable that the rabbit polyclonal anti-Fv1 used in quantitative western blots, which was raised against a purified fragment corresponding to amino acids 20–200 of Fv1NTD, binds more strongly to Fv1MAC than to Fv1SPR1 due to sequence variation in the Fv1NTD. However, this seems highly unlikely since Fv1MAC only differs from Fv1SPR1 at two amino acids within amino acids 20–200 [8].

The relationship between Fv1 concentration and restriction is complex. In some cases, typified by Fv1^n^, restriction properties are unaffected by concentration. Thus, it seems clear that at least some of the novel activities we observed in wild mice [8], e.g. Fv1MAC restriction of EIAV, do not require overexpression. In others, such as NB-MLV restriction by Fv1^b^, restriction only becomes clearly evident following overexpression. Such a relationship may explain the differences in restriction specificities of Fv1MIN2 reported previously [8, 15, 54]. In these studies, restriction of N-MLV, B-MLV but not NB-MLV were observed in cells derived from *M. n. minutoides*, as well as in MDTF cells transduced with the retroviral delivery vector LCNX2 with Fv1MIN2 expression driven by a CMV promoter [15, 54]. In contrast, studies in transduced MDTF cells using the LxIY-Fv1 vector and the two colour FACS assay, also showed restriction of NB-MLV [8]. Here we showed that at lower expression levels, Fv1MIN2 has stronger restriction activities against N-MLV and B-MLV than against NB-MLV (Table 2). Therefore, it is possible that the LxIY-Fv1 vector expresses Fv1MIN2 at a higher level than from LCNX2, thereby allowing the restriction of NB-MLV.

If restriction activities are only fully manifest at overexpression levels, with only partial restriction at induction levels close to that of endogenous Fv1^b^, it seems reasonable to question of the biological significance of such partial restriction activities. Although Fv1 does not appear to be interferon inducible (Vicky Felton, PhD thesis University College London), it is possible that the endogenous levels of some wild mice Fv1s are much higher, therefore allowing full restriction. Since the endogenous level of Fv1^n^ in N-3T3 cells is threefold higher than that of Fv1^b^ in B-3T3 cells (Fig. 2), in some mice the endogenous level may be sufficiently high for full restriction to occur. If overexpression of Fv1 allows the gain of restriction activities, Fv1 may be under evolutionary pressure not only to alter its binding properties to allow recognition of CA from circulating viruses, but also to increase its expression levels, either at the transcriptional level or by enhanced protein stability. It would be interesting to study the any such variation among wild mice, and to ask whether there are adaptations designed to increase the endogenous Fv1 levels, and if so whether there are limits to the expression levels of wild mice Fv1. Alternatively it may be that the combined inhibition exerted by the partial restriction of Fv1 and by other restriction factors such as murine APOBEC3 is sufficient to confer protection against the target virus [55]. To begin to investigate questions associated with Fv1 expression in vivo, we have initiated studies of Fv1 expression in fresh tissues. An initial experiment reveals that expression levels in thymocytes are comparable to that seen in fibroblast lines and rather higher than in splenocytes (Additional file 6).

The biological significance of these partial restriction activities remains open to question. Some of these partial restriction activities might represent a response to a novel threat. As Fv1 evolves to recognise a new emerging virus, it accumulates mutations that lead to the decrease in restriction activity against its old target. It is also possible that some of these Fv1 alleles are under selection from multiple retroviruses simultaneously, and Fv1 has been selected to inhibit all of these viruses, at the cost of weaker restriction against each of these viruses. It has been shown that forced passage of B-MLV in cells expressing Fv1^n^ rapidly leads to the isolation of NB-MLV, while forced passage of N-MLV in Fv1^b^ cells does not [30, 56]. The partial restriction activity against NB-MLV by Fv1^b^ could have an important role in preventing the emergence of NB-tropic virus resulting from adaptation of B-MLV.

## Conclusions

Fv1 can restrict retrovirus replication even when expressed at very low levels, with different alleles showing different restriction specificities. An inducible expression system was established to probe the consequences of factor overexpression. Although novel specificities were revealed with several alleles, in most cases restriction specificity remained unchanged upon overexpression. It thus appears that the activities of Fv1 directed against non-MLVs are manifested at levels equivalent to those that protect inbred mice against MLV. In turn this implies that they have been selected to confer protection against undefined novel viruses.

## Methods

### Plasmids and DNA primers

The pLxIG-Fv1 (formerly designated LFv1IEG) vectors for non-inducible expression of EGFP and wild-type, “mix-and-match” mutant or C-terminal tail deletion mutant of Fv1^n^ and Fv1^b^ have been described previously [5, 6]. The Gateway destination vector pLxIY-DEST (formerly designated pLgatewayIRESEYFP) for non-inducible expression of EYFP and restriction factor has been described previously [57]. The Gateway entry vectors pENTR-Fv1 and delivery vectors LxIY-Fv1 with Fv1^n^, Fv1^b^, Fv1MAC, Fv1MIN2, Fv1SPR1, and Fv1CAR1 ORFs have been previously described [8]. The self-inactivating retroviral vector QCXIX was obtained from ClonTech. pLenti-CMV-rtTA3-Blast (Addgene #26429), pLenti-CMVTRE3G-Puro-DEST (Addgene #27565), and pENTR-LUC (Addgene #17473) were constructed by Eric Campeau [58]. The DNA primers (Sigma) used for PCR and site-directed mutagenesis reactions are listed in Additional file 7.

### Construction of inducible Gateway destination vectors

The inducible Gateway (Life Technologies) destination vectors pTGx-DEST, pTGIx-DEST, pTYx-DEST, pTYIx-DEST were constructed using pQCXIX (ClonTech) as the backbone plasmid. pQCXIX contains the CMV promoter, the 5′ MCS, the EMCV IRES sequence and the 3′ MCS. The TRE3G promoter was amplified by PCR from pLenti-CMVTRE3G-Puro-DEST (Addgene #27565) with the primers TRE3G-F and TRE3G-R, and inserted between ClaI and NotI sites of pQCXIX to replace the CMV promoter. The EGFP sequence was amplified from pLxIG-Fv1^n^ with the primers EGFP-F and EGFP-R, while the EYFP sequence was amplified from pLxIY-DEST using the same primers, both were inserted between NotI and EcoRI sites of the 5′ MCS of pQCXIX. To construct pTGIx-DEST and pTYIx-DEST, the blunt Gateway DEST cassette was obtained by digesting pLenti-CMVTRE3G-Puro-DEST with EcoRV, and inserted into the EcoRV site in the 3′MCS of pQCXIX. To construct pTGx-DEST and pTYx-DEST, the DEST cassette was inserted between the EcoRI site in 5′MCS and the EcoRV site in the 3′MCS of pQCXIX.

### Cloning of inducible delivery vectors

Sequences for Fv1^n^, Fv1^b^ and “mix and match” mutants were amplified from pLxIG-Fv1 vectors by PCR using the forward primer TOPO-Fv1-F which introduces the 5′ CACC sequence, and one of the reverse primers: TOPO-Fv1n-R for Fv1bbn, Fv1bnn and Fv1nbn; TOPO-Fv1b-R for Fv1nnb, Fv1nbn and Fv1bnb; and TOPO-Fv1-notail-R for Fv1nn_ and Fv1bb_. PCR products with Fv1 ORFs were inserted into the pENTR-D-TOPO vector (Life Technologies) by TOPO reaction, in order to obtain pENTR-Fv1 entry vectors. Entry vectors for Fv1^n^ and Fv1^b^ mutants were used in LR reactions with LR clonase (Life Technologies) to insert the Fv1 ORF into either pTGx-DEST or pTGIx-DEST vectors. Similarly, Fv1^b^ and wild mice Fv1 ORFs in entry vectors were inserted into either pTYx-DEST or pTYIx-DEST vectors. To obtain the mutant delivery vector pTGx-Fv1^b^-AGG, the Fv1^b^ ORF with mutated start codon was amplified by PCR using the primers TOPO-AGG-Fv1-F and TOPO-Fv1b-R, inserted into pENTR-D-TOPO by TOPO reaction, and finally inserted to pTGx-DEST by LR reaction. pLenti-CMVTRE3G-Puro-LUC was generated by inserting the Firefly Luciferase ORF from pENTR-LUC into pLenti-CMVTRE3G-Puro-DEST by LR reaction.

### Site directed mutagenesis

To introduce the TAATAA double stop codon into EGFP ORF of pTGx-Fv1^b^, a point mutation was generated by site directed mutagenesis using the primers TGx-TAATAA-F and TGx-TAATAA-R. Mutation of the Fv1 start codon of pLxIG-Fv1^n^ was carried out by site directed mutagenesis using the primers pLxIG-Fv1n-AGG-F and pLxIG-Fv1n-AGG-R. Each 50 μL QuikChange site directed mutagenesis PCR reaction contained 50 ng of plasmid template, 10 pmol of forward primer, 10 pmol of reverse primer, 200 μM of each dNTP, 2.5 U of PfuUltra high fidelity polymerase (Agilent Technologies) in the supplied buffer. PCR reaction was performed at 95 °C for 5 min; followed by 12 cycles of 95 °C for 1 min, 55 °C for 1.5 min, and 68 °C for 15 min; and finally 68 °C for 15. Template DNAs were digested with 30 U of DpnI (Roche) at 37 °C for 2 h, and the amplified mutant plasmids were concentrated by ethanol precipitation before transforming XL10 Gold ultracompetent cells (Agilent Technologies). The introduction of mutations was verified by sequencing [2].

### Cells and virus production

N-3T3, B-3T3, MDTF, MDTF-R18, and 293T cells were maintained in DMEM containing 10 % foetal calf serum and 1 % penicillin and streptomycin. Preparation of retroviral or lentiviral vectors were carried by transient transfection of 293T with 3 plasmids providing Env, Gag-Pol and genome functions, as previously described [5, 57]. Delivery retroviral vectors were prepared by co-transfecting pcz-VSVG, pHIT60, and a retroviral vector for expression of Fv1 and either EGFP or EYFP. Delivery lentiviral vectors were prepared by co-transfecting pcz-VSVG, p8.91, and a lentiviral vector with either blasticidin or puromycin resistance gene. MLV tester viruses were generated by co-transfection of pcz-VSV-G, either pczCFG2fEGFPf for EGFP tester or pczCFG2fEYFPf for EYFP tester, and either pCIGN for N-MLV, pCIGB for B-MLV, or pHIT60 for NB-MLV [5, 57]. EIAV tester viruses were made by co-transfection of pczVSV-G, pONY3.1 and pONY8.4ZCG or pONY4.1Z [59], while FFV tester viruses were produced with pciSFV-1envwt and pcDWF003 [60]. MLV was frozen in aliquots at −80 °C, while EIAV and FFV were freshly prepared for each experiment.

### Generation and screening of MDTF cells expressing rtTA3

MDTF cells were transduced at a MOI less than 0.1 with a lentiviral vector made using the pLenti-CMV-rtTA3-Blast plasmid, for the expression of rtTA3 and the blasticidin resistance gene. Transduced cells were selected with 10 μg/mL blasticidin (Sigma), and single-cell clones were picked from the selected population. To test the leakiness and induction of the *P*_TRE3G_ promoter in these cells, each clone was transduced at a MOI <0.1 with a lentiviral delivery vector made using the pLenti-CMVTRE3G-Puro-LUC vector, which allows the expression of the firefly luciferase gene under the *P*_TRE3G_ promoter, and the expression of puromycin resistance gene under a separate promoter. After selection with 10 µg/mL puromycin, cells were treated with or without 1 μg/mL doxycycline for 24 h. Cells were lysed with the supplied lysis buffer before analysis of luciferase activity using the Luciferase Assay System (Promega) and the Synergy 2 plate reader (Biotek). Relative luminescence signal was normalised to the cell count to obtain normalised relative luciferase activity. Luciferase activity was normalised against cell count from duplicated wells. The MDTF-R18 clone was selected for its low leakiness and high induction, and was used for restriction analyses in this study.

### Restriction assays

The procedure for studying restriction activity was modified from the transient two colour FACS assay previously developed for non-inducible vectors [5, 57]. Briefly, MDTF-R18 cells were transduced with one of the retroviral delivery vectors for the expression of Fv1 and either EGFP or EYFP. 72 h post-transduction, transduced cells were reseeded and incubated with up to 1000 ng/mL doxycycline in DMEM for 24 h, before infection with tester viruses. 10 µg/mL doxycycline was added to each well at 24 h post-infection, and cells were subjected to FACS analysis using FACSVerse, LSR II or LSRFortessa X-20 (BD) flow cytometers at 72 h post-infection. The percentage of tester-positive cells in the Fv1-positive population and in the Fv1-negative population were determined and compared using the FlowJo analysis software, in order to calculate the restriction ratios. Ratios of <0.3 were taken as full restriction; ratios between 0.3 and 0.7 were taken to represent partial restriction; while ratios greater 0.7 were taken to represent no restriction. For restriction assays in cells with superexpression of Fv1, MDTF-R18 cells were transduced with different volume of retroviral delivery viruses or control viruses expressing EGFP. At 72 h post-transduction, transduced cells were mixed with equal number of untransduced cells and reseeded for infection with EYFP tester viruses on the next day. FACS analyses were carried out at 72 h post-infection.

### Quantitative western blots

MDTF-R18 cells transduced with retroviral delivery vectors were incubated with up to 1000 ng/mL doxycycline for 24 h, before cells were harvested and frozen at −80 °C as cell pellets. Frozen N-3T3, B-3T3, and transduced MDTF-R18 cells were lysed in 100 µL RIPA buffer (Thermo Fisher) supplemented with complete EDTA-free protease inhibitors (Roche) at 4 °C for 30 min, clarified by centrifugation at 4 °C for 10 min, before determination of total protein concentrations by BCA protein assay (Thermo Fisher). Lysate samples were boiled in loading buffer containing 2 % SDS and 2.5 % β-mercaptoethanol. Typically 25 µg of lysate was loaded into each well of a 1.5 mm 10 % polyacrylamide gel, along with 5 µL per well of known quantity of recombinant Fv1NTD (20–200) protein [36]. After SDS-PAGE, proteins were transferred to Immobilon-FL PVDF membrane (Merck Millipore) using the Trans-Blot SD semi-dry transfer cell (Bio-rad). Membranes were blocked with 5 % non-fat dry milk in PBS (PBS-milk) at 4 °C overnight. Fv1 was detected using a rabbit polyclonal primary antibody (in house #6689) raised against purified fragment of the Fv1NTD (20–200) [36]. The primary antibody was diluted 1 in 1000 in PBS-milk, and incubated with the membrane for 1 h at room temperature, before washing 4 times with PBS containing 0.1 % Tween-20 (PBS-T). The membrane was then incubated with the IRDye800CW-conjugated goat polyclonal secondary antibody diluted 1 in 5000 in PBS-milk supplemented with 0.01 % SDS for 1 h at room temperature, before washing 4 times with PBS-T. After a final wash with PBS, the membrane was scanned using the Odyssey infrared fluorescent imaging system (LI-COR). Fluorescent intensity of Fv1 and Fv1NTD bands were measured using the ImageStudio software (LI-COR). The quantity of Fv1 in each sample was determined by interpolation of the measured signal of each Fv1 band to a standard curve generated using fluorescent signals from known amount of Fv1NTD on the same blot. In samples of transduced MDTF-R18 cells, only a fraction of cells were transduced with the retroviral delivery virus. Therefore, the amount of Fv1 in the transduced population was calculated by dividing the total quantity of Fv1 in each sample by the proportion of Fv1-positive cells.

## Additional files

10.1186/s12977-016-0276-7 Screening of MDTF single cell clones expressing rtTA3.
10.1186/s12977-016-0276-7 Determination of the concentration of doxycycline required foroptimal GFP separation.
10.1186/s12977-016-0276-7 Timing for the addition of the final high dose of doxycycline.
10.1186/s12977-016-0276-7 Data from quantitative western blot analysis of Fv1n and Fv1b expression levels in N-3T3, B-3T3 and transduced MDTF-R18 cells.
10.1186/s12977-016-0276-7 Data from quantitative western blot analysis of Fv1 expression levels in transduced MDTF-R18 cells.
10.1186/s12977-016-0276-7 Expression of Fv1 in fresh tissues.
10.1186/s12977-016-0276-7 Primers used in this study.
