# Supplementary material for: Expression levels of Fv1: effects on retroviral restriction specificities
Source: Retrovirology. 2016 Jun 24;13:42. doi: 10.1186/s12977-016-0276-7 (PMC4921018; doi:10.1186/s12977-016-0276-7)
Supplement: Supplementary file 1 — 10.1186/s12977-016-0276-7 Screening of MDTF single cell clones expressing rtTA3. [file 12977_2016_276_MOESM1_ESM.pdf]

Additional File 1. Screening of MDTF single cell clones expressing rtTA3.

(A-B) Promoter activity of P_{TRE3G} in single-cell clones expressing the transactivator rtTA3. Cells from each clone were transduced at MOI < 0.1 with a lentiviral delivery vector that allows the expression of the firefly luciferase gene under the PTRE3G promoter, and the expression of puromycin under a separate promoter. After selection with puromycin, cells were treated in the (A) absence or (B) presence of 1μg/ml doxycycline for 24h. (A) Neat lysate from uninduced samples were used for luciferase assays. (B) Lysate samples from induced samples were diluted 1 in 200 before luciferase assay. Relative luminescence signal was normalised to the cell count to obtain normalised relative luciferase activity. Clone 48 represents a negative control from MDTF cells not transduced with the rtTA3 vector virus. The MDTF-R18 clone (highlighted in red) was selected for its low leakiness and high induction, and was used for restriction analyses in this study. (C) MDTF-R18 cells were induced at different concentrations of Dox before analysis by luciferase assay. The graph showed mean and absolute deviation values from duplicate samples in the same experiment.

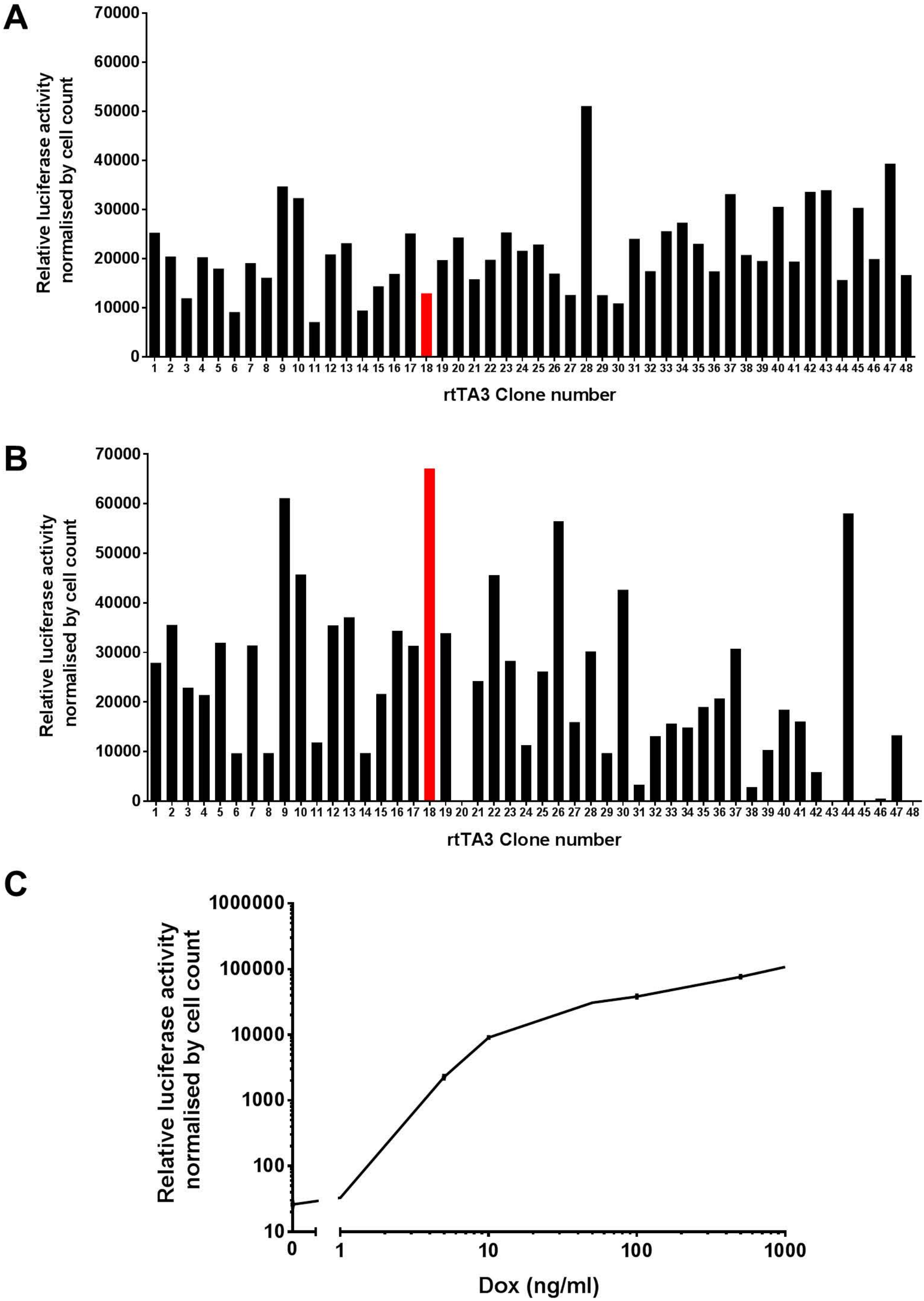
A
Relative luciferase activity normalised by cell count
70000
60000
50000
40000
30000
20000
10000
0
1 2 3 4 5 6 7 8 9 10 11 12 13 14 15 16 17 18 19 20 21 22 23 24 25 26 27 28 29 30 31 32 33 34 35 36 37 38 39 40 41 42 43 44 45 46 47 48
rtTA3 Clone number
B
Relative luciferase activity normalised by cell count
70000
60000
50000
40000
30000
20000
10000
0
1 2 3 4 5 6 7 8 9 10 11 12 13 14 15 16 17 18 19 20 21 22 23 24 25 26 27 28 29 30 31 32 33 34 35 36 37 38 39 40 41 42 43 44 45 46 47 48
rtTA3 Clone number
C
Relative luciferase activity normalised by cell count
1000000
100000
10000
1000
100
10
0
1
10
100
1000
Dox (ng/ml)
