# Supplementary material for: Expression levels of Fv1: effects on retroviral restriction specificities
Source: Retrovirology. 2016 Jun 24;13:42. doi: 10.1186/s12977-016-0276-7 (PMC4921018; doi:10.1186/s12977-016-0276-7)
Supplement: Supplementary file 2 — 10.1186/s12977-016-0276-7 Determination of the concentration of doxycycline required foroptimal GFP separation. [file 12977_2016_276_MOESM2_ESM.pdf]

Additional File 2. Determination of the concentration of doxycycline required for optimal GFP separation.

MDTF-R18 cells previously transduced with TGx-$Fv1^b$ were induced with different concentrations of Dox for 24h before analysis by FACS.

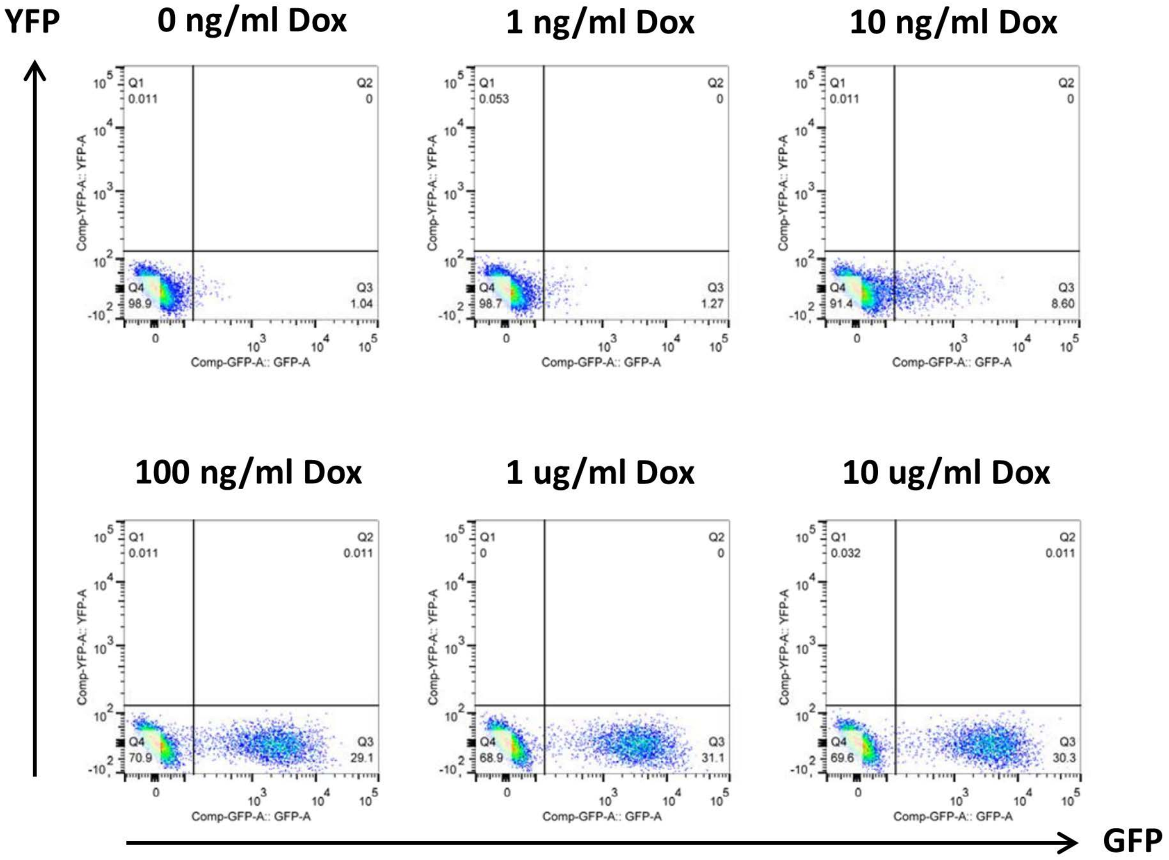
YFP
0 ng/ml Dox
1 ng/ml Dox
10 ng/ml Dox
100 ng/ml Dox
1 ug/ml Dox
10 ug/ml Dox
GFP
Q1 0.011
Q2 0
Q4 98.9
Q3 1.04
Q1 0.053
Q2 0
Q4 98.7
Q3 1.27
Q1 0.011
Q2 0
Q4 91.4
Q3 8.60
Q1 0.011
Q2 0.011
Q4 70.9
Q3 29.1
Q1 0
Q2 0
Q4 68.9
Q3 31.1
Q1 0.032
Q2 0.011
Q4 69.6
Q3 30.3
Comp-YFP-A:: YFP-A
Comp-GFP-A:: GFP-A
