# Supplementary material for: Expression levels of Fv1: effects on retroviral restriction specificities
Source: Retrovirology. 2016 Jun 24;13:42. doi: 10.1186/s12977-016-0276-7 (PMC4921018; doi:10.1186/s12977-016-0276-7)
Supplement: Supplementary file 3 — 10.1186/s12977-016-0276-7 Timing for the addition of the final high dose of doxycycline. [file 12977_2016_276_MOESM3_ESM.pdf]

Additional File 3. Timing for the addition of the final high dose of doxycycline.

Cells transduced with TGx-$Fv1^b$ vector were incubated for 24h in DMEM containing 0 ng/ml (filled circles) or 1000 ng/ml (filled squares) doxycycline, before infection with N-MLV EYFP tester. 10 µg/ml doxycycline was added at different time points from 1 to 24h post-infection. FACS analysis was carried out at 72h post-infection, the restriction ratio was calculated and plotted.

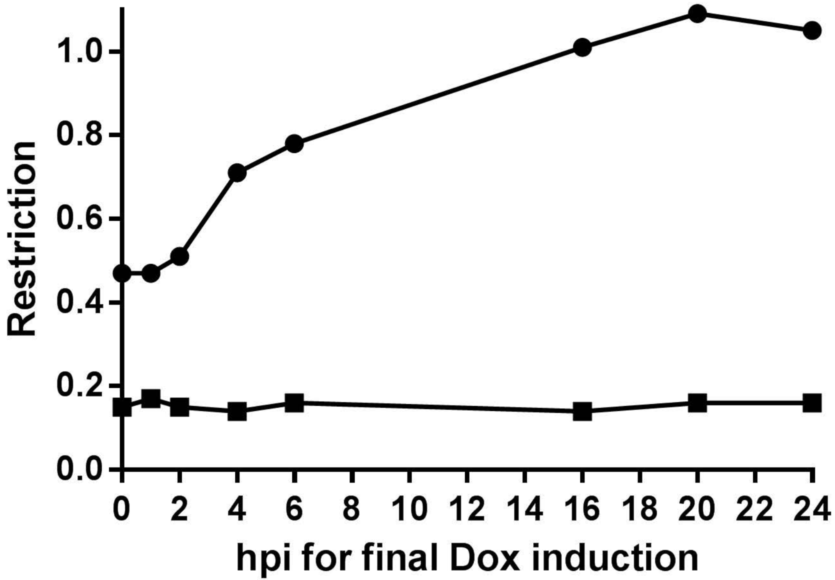
Restriction
1.0
0.8
0.6
0.4
0.2
0.0
0
2
4
6
8
10
12
14
16
18
20
22
24
hpi for final Dox induction
