# Supplementary material for: Expression levels of Fv1: effects on retroviral restriction specificities
Source: Retrovirology. 2016 Jun 24;13:42. doi: 10.1186/s12977-016-0276-7 (PMC4921018; doi:10.1186/s12977-016-0276-7)
Supplement: Supplementary file 4 — 10.1186/s12977-016-0276-7 Data from quantitative western blot analysis of Fv1n and Fv1b expression levels in N-3T3, B-3T3 and transduced MDTF-R18 cells. [file 12977_2016_276_MOESM4_ESM.pdf]

Additional file 4. Data from quantitative western blot analysis of Fv1^n and Fv1^b expression levels in N-3T3, B-3T3 and transduced MDTF-R18 cells.

		Sample	A		B	C	D			
		Western blot	A1	A2	B1	C1	D1	D2		
		[Lysate] ug/ml	1000	1000	1000	1000	3000	3000		
Cell Line	Vector	Dox (ng/ml)	Fv1 quantity relative to Fv1^n in N-3T3 sample						Mean	Count
N-3T3	None	0	1.0	1.0	1.0	1.0	1.0	1.0	1.0	6
B-3T3		0	0.3	0.4	u.d.l.	0.4	u.d.l.	u.d.l.	0.3	3
MDTF-R18	TGx-Fv1^n	0	n.d.	n.d.	u.d.l.	0.8	u.d.l.	u.d.l.	0.8	1
		1000	n.d.	n.d.	u.d.l.	0.8	0.3	0.3	0.4	3
	TGIx-Fv1^n	0	n.d.	n.d.	u.d.l.	1.1	u.d.l.	u.d.l.	1.1	1
		1000	n.d.	n.d.	52.5	35.0	16.0	15.0	29.6	4
	LxIG-Fv1^n	0	43.3	31.1	116.9	86.5	27.6	30.2	55.9	6
	TGx-Fv1^b	0	n.d.	n.d.	u.d.l.	0.1	u.d.l.	0.3	0.2	2
		1000	n.d.	n.d.	u.d.l.	1.1	0.2	0.4	0.6	3
	TGIx-Fv1^b	0	n.d.	n.d.	u.d.l.	1.1	u.d.l.	0.5	0.8	2
		1000	n.d.	n.d.	11.3	11.6	4.9	5.4	8.3	4
	LxIG-Fv1^b	0	9.4	8.3	25.0	23.3	6.8	8.0	13.5	6

n.d. - not determined
u.d.l. - under detection limit
