# Supplementary material for: Expression levels of Fv1: effects on retroviral restriction specificities
Source: Retrovirology. 2016 Jun 24;13:42. doi: 10.1186/s12977-016-0276-7 (PMC4921018; doi:10.1186/s12977-016-0276-7)
Supplement: Supplementary file 5 — 10.1186/s12977-016-0276-7 Data from quantitative western blot analysis of Fv1 expression levels in transduced MDTF-R18 cells. [file 12977_2016_276_MOESM5_ESM.pdf]

Additional file 5. Data from quantitative western blot analysis of Fv1 expression levels in transduced MDTF-R18 cells.

	Sample	A	A	B	B			
	Western Blot	A1	A2	B1	B2			
	[Lysate] ug/ml	1000	3000	1000	1000			
Vector	Dox (ng/ml)	Fv1 quantity relative to LxIY-Fv1[b]				Mean	Std Dev	Count
TYIx-Fv1[b]	0	u.d.l.	u.d.l.	u.d.l.	u.d.l.	u.d.l.		
	10	u.d.l.	u.d.l.	0.13	0.09	0.11	0.03	2
	100	0.33	0.10	0.62	0.73	0.44	0.28	4
	1000	0.53	0.17	0.80	0.77	0.57	0.29	4
LxIY-Fv1[b]	0	1.00	1.00	1.00	1.00	1.00	0.00	4
TYIx-Fv1MIN2	0	u.d.l.	u.d.l.	0.03	u.d.l.	0.03		1
	10	u.d.l.	0.09	0.30	0.28	0.22	0.12	3
	100	2.90	0.41	1.71	2.87	1.97	1.18	4
	1000	4.71	0.67	1.90	3.37	2.66	1.76	4
LxIY-Fv1MIN2	0	14.74	6.00	5.04	9.82	8.90	4.41	4
TYIx-Fv1SPR1	0	u.d.l.	u.d.l.	u.d.l.	u.d.l.	u.d.l.		0
	10	u.d.l.	0.02	0.21	u.d.l.	0.12	0.13	2
	100	0.75	0.11	0.31	0.20	0.34	0.28	4
	1000	0.42	0.16	0.72	0.39	0.42	0.23	4
LxIY-Fv1SPR1	0	1.41	0.59	0.36	0.51	0.71	0.47	4
TYIx-Fv1MAC	0	u.d.l.	u.d.l.	0.06	u.d.l.	0.06		1
	10	0.94	0.04	0.30	0.27	0.38	0.39	4
	100	5.74	0.36	1.97	2.44	2.62	2.26	4
	1000	5.99	0.48	2.35	2.90	2.93	2.29	4
LxIY-Fv1MAC	0	15.88	4.79	4.64	9.33	8.66	5.28	4
TYIx-Fv1CAR1	0	u.d.l.	u.d.l.	u.d.l.	u.d.l.	u.d.l.		0
	10	u.d.l.	0.06	0.29	0.40	0.25	0.17	3
	100	3.08	0.39	2.60	2.99	2.26	1.27	4
	1000	4.04	0.50	3.13	3.33	2.75	1.55	4
LxIY-Fv1CAR1	0	18.69	4.23	5.31	8.98	9.30	6.58	4

n.d. - not determined   u.d.l. - under detection limit
