# Supplementary material for: Expression levels of Fv1: effects on retroviral restriction specificities
Source: Retrovirology. 2016 Jun 24;13:42. doi: 10.1186/s12977-016-0276-7 (PMC4921018; doi:10.1186/s12977-016-0276-7)
Supplement: Supplementary file 6 — 10.1186/s12977-016-0276-7 Expression of Fv1 in fresh tissues. [file 12977_2016_276_MOESM6_ESM.pdf]

Additional file 6. Expression of Fv1 in fresh tissues.

Spleen and thymus cells prepared from C57BL/6 or B6.C3H-Fv1^{n} mice by mechanical disruption and B-3T3 or N-3T3 cells by trypsinization were lysed with triple detergent buffer (150 mM NaCl, 1% NP40, 0.1% SDS, 0.5% deoxycholate, 50 mM Tris-HCl, pH 8.0) containing protease inhibitor (Roche) and benzonase (Sigma). Protein (25 μg per lane) was run on 4 to 20% gradient polyacrylamide gels (BioRad) for western blotting using 1:2000 dilution of the anti-Fv1 NTD antibody and 1:10,000 dilution of the HRP-conjugated anti-rabbit antibody (Sigma). The bands were visualized using the ECL reagent from Millipore.

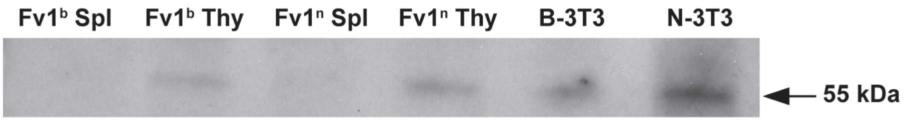
Fv1b Spl
Fv1b Thy
Fv1n Spl
Fv1n Thy
B-3T3
N-3T3
55 kDa
