# Supplementary material for: Expression levels of Fv1: effects on retroviral restriction specificities
Source: Retrovirology. 2016 Jun 24;13:42. doi: 10.1186/s12977-016-0276-7 (PMC4921018; doi:10.1186/s12977-016-0276-7)
Supplement: Supplementary file 7 — 10.1186/s12977-016-0276-7 Primers used in this study. [file 12977_2016_276_MOESM7_ESM.pdf]

Additional File 7 Primers used in this study

Primer	Sequence
TRE3G-F	GTATATATCGATCACGAGACTAGCCTCGAGAG
TRE3G-R	GTATATGCGGCCGCCCACCACACTGGACTAGTC
EGFP-F	GTATATGCGGCCGCCATGGTGAGCAAGGGCGAGG
EGFP-R	GTATATGAATTCTTACTTGTACAGCTCGTCC
TOPO-Fv1-F	CACCATGAATTTCCCACGTGCGCTTG
TOPO-Fv1n-R	TCGGAGTTTTGTAGCTGCTG
TOPO-Fv1b-R	TTAACTGTTGCTTTGATGTTTC
TOPO-Fv1-notail-R	TCAAGCTGCTGTTGGCTTTAAAC
TOPO-AGG-Fv1-F	CACCAGGAATTTCCCACGTGCGC
TGx-TAATAA-F	CGGCATGGACGAGCTGTACAAGTAATAATTATCAACAAG
TGx-TAATAA-R	CTTGTTGATAATTATTACTTGTACAGCTCGTCCATGCCG
pLxIG-Fv1n-AGG-F	CAAAAGTCTGAAGAGGAATTTCCCACGTGC
pLxIG-Fv1n-AGG-R	GCACGTGGGAAATTCCTCTTCAGACTTTTG
